# Comprehensive Prediction Analysis of Novel Noncoding Regulatory Variants Identified in the MicroRNA Binding Regions in Complement System Genes

**DOI:** 10.3390/ijms27135877

**Published:** 2026-06-30

**Authors:** Anthony Shadid, Haydn E. Rich, Kathryn D. Hok, Marie-Francoise Doursout, Marcos I. Restrepo, Nirmal K. Banda, Lavanya Gunamalai, Pooja Shivshankar

**Affiliations:** 1 Institute of Molecular Medicine, UTHealth-McGovern Medical School, Houston, TX 77030, USA; 2 Department of Anesthesiology, UTHealth-McGovern Medical School, Houston, TX 77030, USA; 3 VA-San Antonio Geriatric Research Education and Clinical Center (GRECC), South Texas Veterans Health Care System Audie L. Murphy Division, UTHealth San Antonio, San Antonio, TX 78229, USA; restrepom@uthscsa.edu; 4 Division of Rheumatology, Department of Medicine, School of Medicine, University of Colorado Anschutz Medical Campus, Aurora, CO 80045, USA; nirmal.banda@cuanschutz.edu

**Keywords:** single nucleotide polymorphism (SNP), complement immune system, immune regulation, membrane attack complex, 3′UTR, miRNA binding, post-transcriptional regulation, genes-disease association

## Abstract

The complement system is a central component of innate immunity that coordinates host defense, immune surveillance, and inflammatory responses through tightly regulated proteolytic cascades. Genetic variation within complement genes contributes substantially to interindividual differences in complement activity and disease susceptibility. While coding variants directly alter complement protein structure and function, the microRNA (miRNA)-mediated control and post-transcriptional regulation is not fully understood in shaping complement gene expression across immune and inflammatory mechanisms. Complement pathway genes exhibit extensive and heterogeneous 3′-untranslated regions (3′UTRs), which serve as primary platforms for miRNA binding and RNA-binding protein interactions. Both common and rare single-nucleotide polymorphisms within coding regions and 3′UTRs can influence miRNA targeting efficiency, disrupt regulatory motifs, or alter mRNA turnover, thereby fine-tuning complement activity rather than causing complete loss of function. Here, we systematically analyzed miRNA binding sites and single-nucleotide polymorphisms (SNPs) within the 3′UTRs of complement pathway genes spanning the classical, lectin, alternative, and terminal pathways. Our analysis uncovered heterogeneous patterns of miRNA-mediated regulation across the complement system. While core complement components showed relatively sparse conserved miRNA targeting, regulatory factors, receptors, and terminal pathway proteins emerged as densely regulated nodes, harboring multiple conserved binding sites. A broad repertoire of miRNAs was predicted to engage complement genes in a pathway-specific manner, implicating these interactions in inflammation, cancer progression, metabolic regulation, and immune signaling. SNPs within miRNA-binding regions are predicted to disrupt or create regulatory interactions, providing a mechanistic basis for how non-coding genetic variation can alter gene expression and modulate disease susceptibility. Our findings indicate miRNA-mediated post-transcriptional regulation as an important yet underappreciated layer of complement system control, providing a framework for understanding how regulatory genetic variation shapes complement-driven immune responses and disease risk.

## 1. Introduction

Genetic variation significantly contributes to individual differences in complement system activity. Rare, high-penetrance coding mutations in complement genes are established causes of monogenic disorders such as atypical hemolytic uremic syndrome (aHUS) and hereditary angioedema [1,2]. Concurrently, large-scale studies have uncovered numerous common single-nucleotide polymorphisms (SNPs) in complement loci that affect disease susceptibility and clinical outcomes. These variants comprise both protein-altering coding changes, which directly impact complement protein structure or function, and noncoding variants, which modulate gene expression, transcript processing, or regulatory responsiveness. Collectively, these genetic differences influence the strength, timing, and tissue specificity of complement activation [3,4].

Several complement genes exhibit complex genomic architectures that accommodate both functional coding variation and extensive regulatory control. Genes such as *CFH*, *C3*, *CFB*, *CD46*, and *SERPING1* harbor missense variants with demonstrable effects on complement activity, as well as intronic, promoter, and splice-region variants that may modulate transcript abundance or isoform usage. Advances in genome-wide association studies, expression quantitative trait locus analyses, and functional genomics have begun to clarify how these coding and noncoding variants act independently or in combination to influence complement biology under physiological and pathological conditions [5,6,7].

Despite extensive genetic investigation, the literature remains fragmented, often emphasizing either rare coding mutations or isolated noncoding regulatory variants within specific disease contexts. A unified framework that integrates both classes of genetic variation within the complement system remains lacking. Such an integrative perspective is essential for understanding how structural alterations of complement proteins intersect with transcriptional and post-transcriptional regulation to drive immune dysregulation and disease risk [8,9,10].

Among post-transcriptional regulatory mechanisms, miRNAs have emerged as key modulators of gene expression. miRNAs are short noncoding RNAs that bind to complementary sequences within the 3′UTRs of target mRNAs, leading to translational repression or mRNA degradation [11,12,13]. The specificity of this interaction is largely determined by the miRNA seed region (positions 2–8), making these binding sites particularly sensitive to sequence variation. Consequently, SNPs occurring within miRNA-binding regions, often referred to as miRNA target site polymorphisms (miR-SNPs), may disrupt existing regulatory interactions or generate novel binding motifs, thereby altering gene expression and potentially influencing disease risk.

Despite the central role of complement in immunity and disease, the landscape of miRNA-mediated regulation across complement genes remains incompletely characterized. In particular, the functional impact of genetic variation within miRNA-binding sites in the 3′UTRs of the complement genes has not been systematically characterized. Understanding these interactions may provide important insights into how noncoding variants contribute to dysregulation of the complement system and immune-related diseases. In this study, we performed a comprehensive computational analysis to identify miRNA binding sites within the 3′UTRs of complement pathway genes spanning the classical, lectin, alternative, and terminal pathways, as well as complement regulators and receptors. We further mapped SNPs overlapping predicted miRNA-binding regions and evaluated their potential functional impact using variant annotation resources and functional prediction scores. By integrating rsID-level mapping, miRNA target prediction, and pathway network analyses, our study addresses this gap and provides a systems-level framework to highlight candidate miR-SNPs, linking non-coding variation to complement dysregulation that may contribute to immune-related disease susceptibility.

## 2. Results

### 2.1. Novel MiRNA Regulatory Site Variants in 3′UTR Regions

The potential regulatory impact of genetic variation within complement pathway genes and their 3′UTRs, as the genomic loci of interest, was examined for overlap with predicted miRNA binding sites using the UCSC Genome Browser (GRCh38 assembly), as shown in Table S1 (BLAT results). To systematically evaluate the post-transcriptional regulatory potential across the complement system, full-length 3′UTRs were curated to analyze variant forms including altered single-nucleotide variants and deletion mutations in core complement components, regulators, and receptors spanning the classical, lectin, alternative, and terminal pathways (Table S1). Collectively, we have identified 241 rsIDs comprising these variants in the complement system genes.

**Figure 1:**
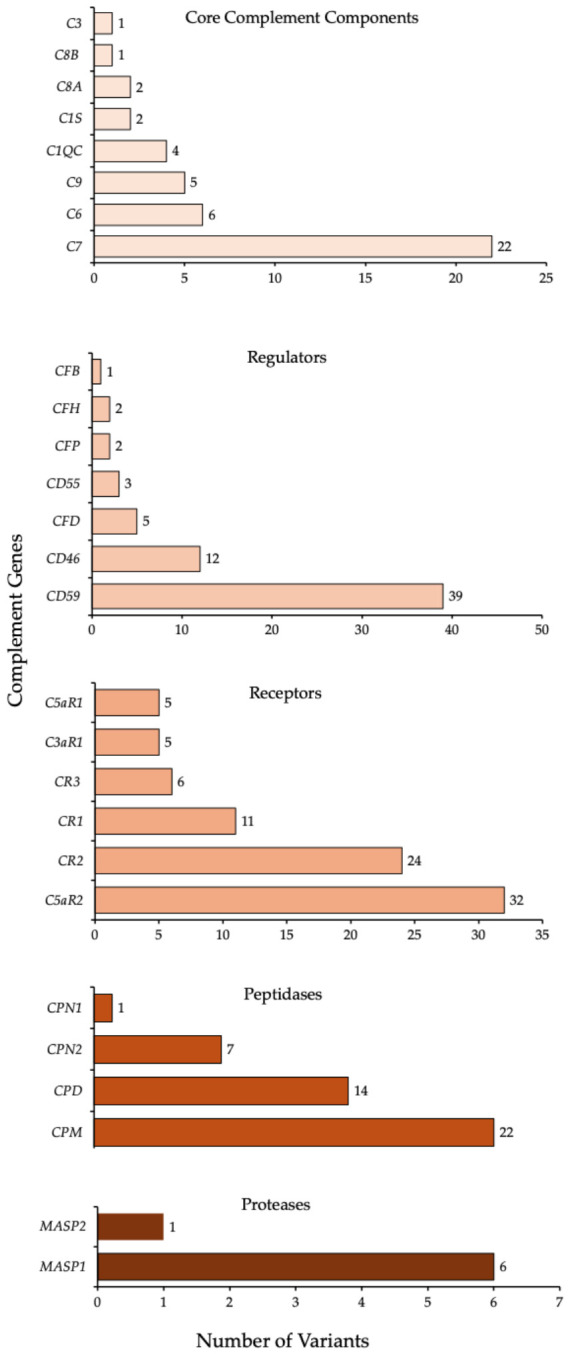
The distribution of SNPs in 3′UTR miRNA binding sites across complement genes. The bar plot shows the number of SNPs identified within predicted miRNA binding sites in the 3′UTRs of complement-related genes. Genes are ordered by total SNP counts. Complement regulatory and enzymatic genes exhibit higher SNP density compared to core cascade components.

Among the analyzed genes, the highest number of SNPs within predicted miRNA binding sites was observed in *CD59* (39 variants), followed by *C5aR2* (32 variants), *CR2* (24 variants), *C7* (22 variants), and *CPM* (22 variants). Several additional complement regulators and receptors also harbored multiple variants, including *CPD* (14 variants), *CD46* (12 variants), and *CR1* (11 variants). Moderate numbers of variants were detected in *MASP1* (7 variants), *CPN2* (7 variants), *C6* (6 variants), and *CR3/ITGAM* (6 variants). Other complement components, such as *C1QC*, *C9*, *CFD*, *C3aR1*, *C5aR1*, and *CFP*, contained fewer variants, ranging from 2 to 5 SNPs. Only a few variants were identified in certain genes, including *C3*, *C8B*, *CFB*, and *CPN1*, each harboring a single SNP within a predicted miRNA-binding region. To our surprise, no variants were identified in the 3′UTR of complement factor *C5*. Overall, these findings demonstrate that genetic variation within miRNA target sites is widely distributed across complement pathway genes, with a higher density observed in complement regulatory proteins and receptors. The distribution of variants of the 3′UTR regions is illustrated in the bar graph (Figure 1).

### 2.2. Novel Predicted miRNAs Binding in the 3′UTRs of Complement Factors

**Figure 2:**
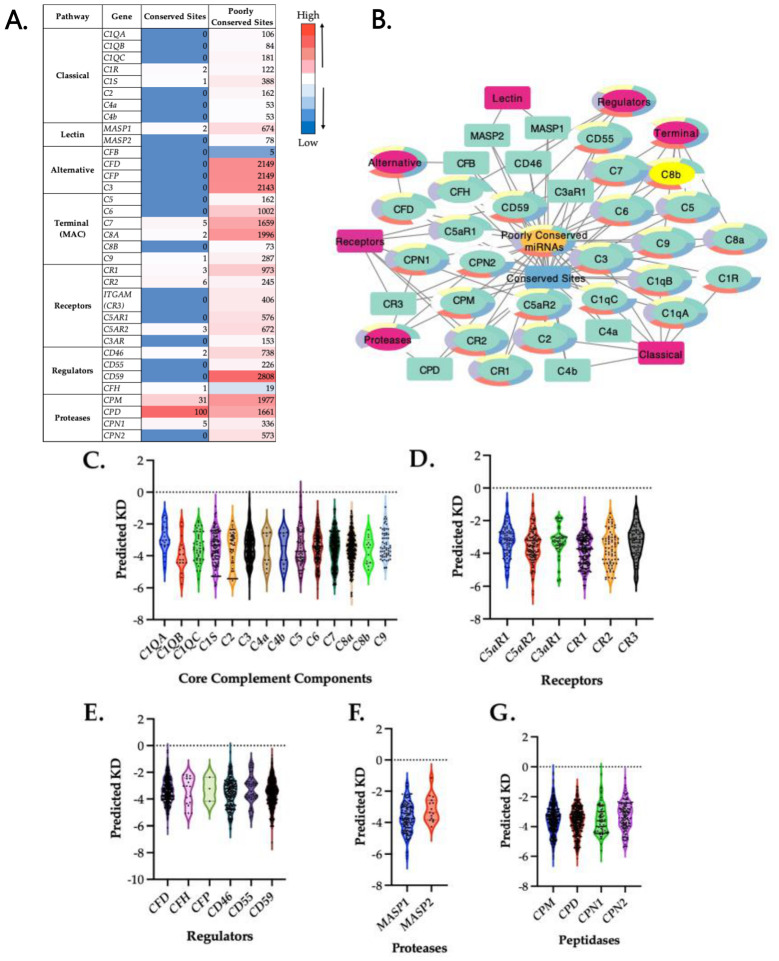
miRNA-mediated regulatory landscape of complement pathwa y compon ents and dis tribution of expression across conditions. (**A**). Heatmap summarizing predicted miRNA targeting of complement pathway genes. Genes are grouped by functional pathways, including Classical, Lectin, Alternative, Terminal (MAC), Receptors, Regulators, and Proteases. The “Predicted miRNA sites” column (red gradient) reflects the relative abundance of predicted miRNA binding sites per gene (low to high), while the “Conserved sites” column (blue gradient) indicates the degree of evolutionary conservation of these sites (low to high). (**B**). Integrated KEGG, Reactome, and WikiPathways network showing complement pathway components and their association with conserved and poorly conserved miRNA-binding sites in the 3′UTRs. Nodes represent complement genes grouped into the classical, lectin, alternative, terminal, receptor, protease, and regulatory pathways. Central nodes labeled “Conserved Sites” and “Poorly Conserved miRNAs” indicate predicted miRNA interactions identified within target 3′UTRs. Highly connected hub genes, particularly C3 and C5, exhibit extensive interactions across multiple complement branches, suggesting widespread miRNA-mediated control mechanisms. (**C**–**G**). Violin plots depicting the distribution of the predicted KD values as miRNA binding efficiency across the complement system genes. Each violin represents a distinct abundance of novel predicted miRNAs binding sites in the 3′UTR regions of complement components listed in different categories.

Comprehensive analysis by TargetScan 8.0 of complement pathway genes revealed conserved as well as poorly conserved miRNA binding sites in the 3′UTRs across all major complement activation pathways (Figure 2; Tables S2 and S3). Heatmap analysis demonstrated unequal distribution of miRNA binding to the 3′UTRs of the complement factors, whereas the miRNA binding was enriched in key effector molecules, including *C3*, *CFD*, *CFP*, *C8a, CD59*, and *CPD*, as well as inflammatory receptors such as *C3aR*, *C5aR1*, and *C5aR2*. The presence of numerous conserved miRNA target sites across complement genes suggests that these regulatory interactions may be evolutionarily maintained (Figure 2A).

Enrichment network analysis across KEGG, Reactome, and WikiPathways consistently showed that complement activation and regulation are the most prominent biological processes in the gene set (Figure 2B). Complement and coagulation cascades represented the top KEGG pathway (*p* = 4.93 × 10^−55^). Reactome analysis revealed enrichment of complement activation, regulation, C3/C5 activation, and terminal pathway modules. WikiPathways identified the complement system in neuronal development and plasticity as the most enriched pathway (*p* = 1.13 × 10^−52^). Complement genes were additionally associated with infectious diseases, COVID-19, systemic lupus erythematosus (SLE), allograft rejection, oxidative stress, and acute inflammatory responses. These findings indicate that the complement genes targeted by conserved and poorly conserved miRNA binding sites participate in a highly interconnected immune network and suggest that miRNA-mediated post-transcriptional mechanisms may influence complement activity across multiple physiological and pathological processes.

Analysis of predicted miRNA binding affinities revealed heterogeneous KD distributions across all complement pathway components (Figure 2C–G). Differences in distribution shape, variability, and central tendency indicated the presence of high- and low-affinity miRNA interaction strengths. Terminal pathway genes and complement receptors showed particularly wide distributions, suggesting extensive post-transcriptional regulation, thereby modulating complement-driven inflammation and immune responses.

### 2.3. Computational Modeling of the 3′UTR Variants

**Figure 3:**
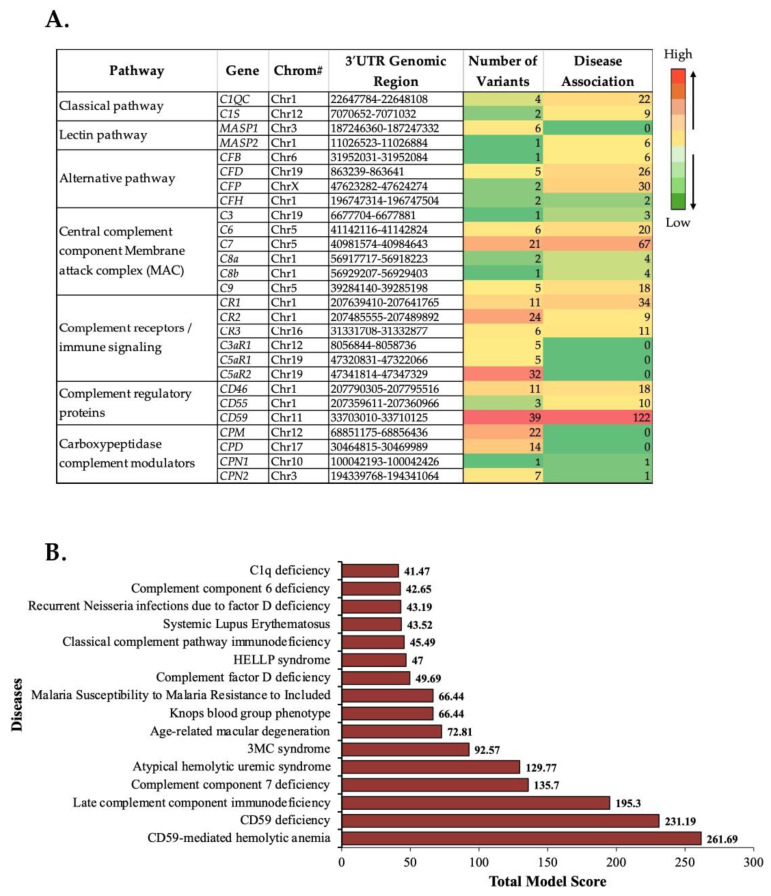
Integrated heatmap and gene-disease association analysis of complement genes containing SNPs in 3′ UTR miRNA binding regions. (**A**). Heatmap of relative genetic variation and disease association scores across the analyzed gene set. Each row represents an individual gene and includes its chromosomal location (chromosome number, #) and the 3′UTR sequence region. The two columns correspond to the Variant Score and Disease Score. Color intensity ranges from green (low) to red (high), reflecting the relative magnitude of each score, as indicated by the accompanying color scale. (**B**). The miR-SNPs of complement system genes were evaluated for functional impact using Combined Annotation Dependent Depletion (CADD) PHRED scores. Variant-level disease associations were aggregated at the gene level to construct gene-disease interaction matrices, which were visualized using bagplot. The analysis highlights complement genes with potential regulatory variation that may influence disease susceptibility.

Distinct complement genes contribute to specific clinical phenotypes through mechanisms that include single-nucleotide substitutions and complex multi-allelic structural variation in the coding sequence regions [14,15,16,17]. However, the functional significance of non-coding variation within the 3′UTRs of complement genes has received comparatively little attention. Therefore, a comprehensive rsID-level mapping of the 3′UTRs of complement and immune regulatory genes revealed a highly structured and non-random architecture of disease-associated variation (Figure 3, Table S4). The model incorporated 330 unique regulatory 3′UTR variants across complement components, receptors, and regulatory genes. After excluding nonspecific or quantitative traits, the filtered dataset contained 434 variant–disease associations spanning 40 distinct disease categories (Table S4). Disease-linked variants were unevenly distributed across genes, with enrichment in regulators of terminal complement activity and of the alternative pathway. Composite scores ranged across the model spectrum (mean 4.4), with higher values reflecting stronger support from multiple independent evidence sources, greater population rarity, and higher predicted functional impact. The highest-scoring associations were concentrated in genes including CD59, C7, CFH, CFB, and CD46, consistent with their established roles in complement-mediated pathology. Variants in CD59 and C7 were associated primarily with complement deficiency phenotypes, whereas variants in CFH, CFB, and CD46 showed associations with aHUS and age-related macular degeneration (AMD). These genes contributed disproportionately to the overall disease burden due to both higher variant density and stronger supporting annotations. Overall, the model indicates that regulatory polymorphisms within complement genes, particularly those located in predicted microRNA-binding regions, cluster within specific disease categories and may influence disease susceptibility through modulation of gene expression rather than protein-coding alterations (Figure 3, Table S4).

As detailed in Table S4, C1QC and C1S display multi-allelic and indel-rich variation associated with classical complement pathway immunodeficiency (C1q deficiency), exemplified by rs10601144, which exhibits extensive allelic complexity (–/T/TT/TTTTTTT). These results suggest that early complement components are particularly sensitive to structural genomic variation, likely because of their roles in immune complex recognition and the initiation of complement activation. In contrast, terminal pathway genes (C6–C9) exhibit relatively sparse, low-frequency variants, except for potential C6 deficiency indicated by rs150777213 (A > C), suggesting reduced allelic diversification in the MAC genes compared to upstream components.

Further, pronounced locus-specific clustering is observed in MASP1, where multiple rsIDs, including rs72549274 (G/C > A), rs78625548 (A > G), rs16848736 (C > T), rs137929458 (PolyT > –/T/TT), rs3836477 (PolyT > –/T), and rs698090 (T > C), are exclusively associated with 3MC syndrome, which is a rare autosomal recessive disorder observed in patients carrying functional mutations in *MASP1* gene along with other lectin pathway *COLEC10/11* [18]. This pattern suggests that lectin pathway dysregulation is closely linked to developmental pathology and that *MASP1* may serve as a structural hotspot for allelic diversity in the 3′UTR.

In contrast, genes of the alternative complement pathway, particularly *CFH* and *CFB*, exhibit clear pleiotropic effects. Shared variants such as rs4151672 (G/T > C), rs35742764 (T > C), and rs488738 (A/G > T) contribute to both AMD and aHUS [19,20]. This overlap supports the hypothesis that dysregulation of complement activation at the level of C3 convertase control constitutes a central mechanistic axis linking renal microangiopathy and retinal degeneration. The presence of multiple ClinVar-annotated “benign” variants indicates incomplete clinical interpretation and highlights the importance of context-dependent pathogenicity in complement genetics (Table S4).

*CD59* demonstrates a striking enrichment of allelic diversity, with an extensive series of variants, including rs79098693 (A/G/T > C), rs77695144 (A/C > G), rs41275158 (T > C), rs75265524 (G > A), rs113743057 (T > C), rs77762316 (G > T), rs2231461 (G > C), rs77171493 (A > G), rs7123070 (A > T), rs74352141 (C), rs73482903 (A), rs73482912 (G), rs74580548 (C), rs77354123 (A), rs73481002 (G), rs12272660 (C), rs1047581 (C), rs842 (T), rs11585 (A), rs11032349/rs11032350 (C/T), rs560607574 (-/A/AA/AAA), rs58634493 (-), and rs7046/rs7357/rs7130936/rs704697 (multi-allelic forms), consistently associated with CD59 deficiency and CD59-mediated hemolytic anemia. This pattern indicates a highly mutation-prone, structurally unstable locus, likely influenced by repetitive sequences, copy-number variation, and regulatory disruption. In contrast to CFH [19] or CD46 [21,22], where variation is more dispersed, CD59 exhibits dense clustering of functionally convergent alleles, suggesting a dosage-sensitive gene with limited tolerance to perturbations in membrane complement regulation (Table S4).

The immune receptor genes CR1, CR2, and ITGAM exhibit a distinct evolutionary signature, characterized by dual associations with SLE and with infectious disease susceptibility or resistance (malaria, Knops blood group phenotype). Variants such as rs57994498 (C), rs7555024 (T), rs4844385 (A/C/G), and rs10779339 (C/G) in CR1 reflect long-standing selective pressure, likely resulting from host–pathogen co-evolution in malaria-endemic regions. Similarly, ITGAM variants (rs9933520, rs4597342, rs58384006) underscore its role as a key mediator of leukocyte adhesion and immune tolerance, with pleiotropic effects on autoimmunity dataset reveals a hierarchical organization of complement genetics: (i) structural hotspot genes (CD59, MASP1, C1QC) characterized by dense multi-allelic variation, (ii) pleiotropic regulatory nodes (CFH, CFB, CD46) bridging multiple disease phenotypes, and (iii) evolutionarily constrained immune receptors (CR1, ITGAM) shaped by infectious disease selection. Together, these patterns support a unified model in which complement-mediated diseases arise not from isolated variants, but from distributed genetic perturbations across pathway nodes with varying degrees of structural constraint, functional redundancy, and evolutionary pressure (Table S4**)**.

### 2.4. SNPs of the Coding Sequences of Complement Factors

Previously published studies have demonstrated that numerous missense, nonsense, and frameshift variants in genes encoding complement components and regulators of complement activation were associated with a broad spectrum of human diseases, including complement deficiency syndromes [23,24,25], recurrent infections [26,27], aHUS [21,28,29,30,31,32], SLE [33,34,35], AMD [14,20,36] inflammatory renal diseases [37], complement hyperactivation and CHAPLE disease [38,39], hemolytic anemia [40], and periodontal Ehlers–Danlos syndrome [41,42]. Several common polymorphisms were linked to altered complement activity and disease susceptibility or protection [14,22], whereas truncating and loss-of-function variants frequently resulted in impaired complement activation or dysregulated complement control [15]. Collectively, these findings demonstrate that pathogenic variation is distributed across all major complement pathways and their regulators, highlighting the contribution of coding sequence variants to complement-mediated disorders. The detailed discussion on these coding sequence genetic variants is presented in the Supplementary Data File S1, containing Tables S5–S9.

## 3. Discussion

The complement system is a central component of innate immunity and plays critical roles in host defense, immune surveillance, and inflammatory regulation. While coding mutations in complement genes are well-established causes of several immune and inflammatory disorders, emerging evidence suggests that noncoding regulatory variants, for example, in CFH, also contribute significantly to complement system variability and disease susceptibility [19]. Therefore, in this study, we integrated genomic annotations with miRNA target prediction to identify SNPs located within the predicted miRNA binding sites across complement pathway genes.

In contrast to the well-characterized coding sequence variants in complement genes that directly disrupt protein structure and function (e.g., CD59 truncating mutations causing MAC dysregulation or CR1 missense variants affecting ligand binding), the 3′UTR SNP landscape represents a largely regulatory layer of variation with subtler, expression-level effects [43,44,45,46]. These non-coding variants are enriched in genes such as CD59, C5aR2, CR2, C7, CPM, CD46, and CFH, and are associated with complex disease phenotypes like aHUS and AMD, likely through modulation of transcript stability and microRNA-mediated post-transcriptional control rather than overt loss of protein function.

As revealed by the pathway enrichment analysis, genes of the complement system appear to be susceptible to miRNA-mediated regulation, as many encode potent inflammatory effector proteins that require stringent expression control to balance host defense with tissue protection. The enrichment analysis also reveals a hierarchical pattern of miRNA regulation within the complement system, characterized by selective targeting of regulatory and terminal pathway components, while core activation proteins are largely devoid of conserved miRNA-binding sites. The absence of conserved miRNA target sites in central complement components, such as C3 and C5, may indicate evolutionary pressure to maintain rapid, robust activation of the innate immune response. These proteins occupy pivotal positions in the cascade, and their regulation is likely controlled predominantly at the transcriptional and proteolytic levels rather than through miRNA-mediated repression. Similar resistance to miRNA regulation has been observed in other essential innate immune genes [47,48]. While no formal evolutionary analysis was performed, the observed distribution of miRNA-binding sites suggests a non-random regulatory architecture that may reflect differential constraints across complement pathway components.

In contrast, upstream proteases (C1R, C1S) and terminal components (C7-C9) exhibit conserved miRNA targeting, indicating that miRNAs may fine-tune complement activation at key regulatory checkpoints. Regulation of the membrane attack complex (MAC) by miRNAs could serve to limit host tissue damage during inflammation, a mechanism consistent with the role of complement in chronic inflammatory diseases and cardiovascular pathology [4]. Complement regulatory proteins, including CD46 and CFP, also show conserved miRNA interactions, highlighting the importance of post-transcriptional control in maintaining self-tolerance. Dysregulation of these proteins has been implicated in autoimmune diseases and atherosclerosis, suggesting that miRNA-mediated modulation may contribute to disease susceptibility [3].

The identification of conserved targeting of MASP1 by miR-122-5p is particularly notable, as miR-122 is a liver-enriched miRNA. Given that many complement proteins are synthesized in hepatocytes, this finding supports tissue-specific regulation of complement activity. Similarly, the targeting of CR2 by the miR-34 family, a key component of the p53 pathway, suggests a potential link between complement signaling, apoptosis, and immune surveillance.

Recurrently targeting miRNAs, such as miR-214-3p, miR-325-3p, and the let-7 family, may function as master regulators of complement activity by coordinating the expression of multiple genes within the pathway. Notably, several inflammation-associated miRNAs identified in this study, including miR-21 and miR-146a, have previously been implicated in immune regulation and cardiovascular disease, further supporting the biological relevance of these predictions [49,50]. Collectively, these findings suggest that miRNA-mediated regulation of the complement system is strategically positioned to modulate immune responses without compromising essential host defense mechanisms.

**Figure 4:**
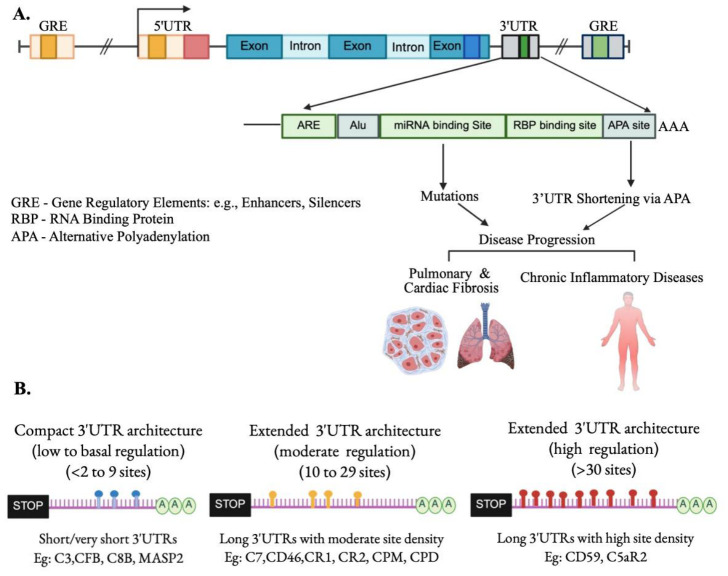
Impact of 3′-UTR regulatory elements and SNPs on gene expression and disease progression. (**A**). Schematic representation of gene architecture highlighting gene regulatory elements (GREs; e.g., enhancers and silencers), the 5′-untranslated region (5′UTR), coding exons and introns, and the 3′-untranslated region (3′UTR). The expanded 3′-UTR region contains key post-transcriptional regulatory elements, including AU-rich elements (AREs), Alu repetitive elements, microRNA (miRNA) binding sites, RNA-binding protein (RBP) binding sites, and alternative polyadenylation (APA) sites. Single-nucleotide polymorphisms (SNPs) within the 3′UTR can disrupt or create miRNA and RBP binding sites, thereby altering mRNA stability and translational efficiency. In addition, APA-mediated 3′UTR shortening can lead to the loss of regulatory elements, resulting in reduced post-transcriptional repression and increased protein expression. These alterations contribute to dysregulated gene expression and are implicated in disease progression, including pulmonary and cardiac fibrosis as well as chronic inflammatory diseases. (**B**). Schematic representations of complement gene mRNAs showing a conserved coding region terminating at a stop codon (STOP), followed by distinct 3′UTRs (purple) and a poly (A) tail (AAA). Colored symbols within the 3′UTR denote regulatory elements such as microRNA binding sites and RNA-binding protein interaction motifs. Transcripts with relatively short, sparsely populated 3′UTRs (**left**) are characteristic of several constitutively expressed complement components (e.g., C3, CFB, etc), which require stable basal expression. In contrast, transcripts with longer and more complex 3′UTRs (**middle** and **right**), enriched for regulatory elements, are representative of complement regulators and inflammation-responsive genes (e.g., C7, CD46, CD59, C5aR2, etc), enabling fine-tuned control of mRNA stability and translation. This schematic highlights how post-transcriptional regulation via 3′UTR diversity precisely modulates complement gene expression during homeostasis and inflammatory responses. Created in BioRender. Shivshankar, P. https://BioRender.com/2o0w4ry (accessed on 11 March 2026).

In addition to miRNA binding, alternative polyadenylation (APA) can further influence the regulatory landscape of these transcripts (Figure 4). APA can generate mRNA isoforms with shorter or longer 3′UTRs, thereby altering the availability of miRNA-binding sites. Shortening of the 3′UTR through APA may remove regulatory regions containing miRNA-binding motifs or associated miR-SNPs, allowing transcripts to escape miRNA-mediated repression. Conversely, longer 3′UTR isoforms may retain these sites and remain susceptible to miRNA regulation (Figure 4A). As a result, the combined effects of APA and miR-SNPs may significantly influence complement gene expression, thereby modulating inflammatory conditions. Emerging evidence indicates that inflammatory signaling pathways can modify APA patterns and reshape the 3′UTR architecture of genes involved in immune responses (Figure 4B) [51,52]. Consistent with this concept, APA-mediated remodeling of 3′UTRs has been observed in fibrotic and inflammatory lung diseases, where shortening of transcripts such as Col1a1 and Has2 contributes to disease progression [53,54]. Because 3′UTRs serve as platforms for miRNA binding, RNA-binding protein interactions, and context-dependent regulation of transcript stability, structural variation in these regions suggests that complement gene expression is finely tuned through multiple layers of post-transcriptional control.

From a translational perspective, these findings suggest that miRNA-complement regulatory interactions could represent potential therapeutic targets. Modulating specific miRNAs that regulate complement inhibitors, such as CD59, may offer a strategy to control complement activation without directly inhibiting complement proteins, potentially reducing adverse immunosuppressive effects. The identification of CD59 as a major regulatory hotspot is particularly noteworthy. CD59 is a membrane-bound complement regulatory protein that inhibits the formation of the membrane attack complex (MAC), thereby protecting host cells from complement-mediated lysis. The high density of miRNA binding sites within the CD59 3′UTR suggests that fine-tuned miRNA regulation may be critical for maintaining appropriate CD59 expression levels during inflammatory responses. The translational potential of these findings will ultimately depend on experimental validation of the predicted miRNA-complement gene interactions in physiologically relevant models [55].

## 4. Materials and Methods

### 4.1. Gene Selection and Genomic Data Retrieval

Complement system genes representing the classical, lectin, alternative, and terminal pathways, along with regulatory proteins and receptors, were selected based on established literature [56,57,58]. These included complement components (C1QA, C1QB, C1QC, C1R, C1S, C2-C9), regulatory factors (CFH, CFI, CD46, CD55, CD59), alternative pathway proteins (CFB, CFD, CFP), complement receptors (CR1, CR2, ITGAM/CR3, C3aR1, C5aR1, C5aR2), and carboxypeptidases (CPM, CPD, CPN1, CPN2). Genomic coordinates, gene structure, and full-length 3′ untranslated region (3′UTR) sequences were obtained (Table S1) from the UCSC Genome Browser (GRCh38/hg38 assembly).

### 4.2. SNP Annotation and Predicted Regulatory Elements Mapping

Single-nucleotide polymorphisms (SNPs) located within the 3′-UTRs of complement genes were identified using publicly available variant databases and mapped to genomic coordinates obtained from the UCSC Genome Browser. Predicted regulatory elements within the 3′ untranslated regions (3′UTRs) of complement pathway genes were systematically mapped using genomic coordinate-based annotation. For each gene, 3′UTR regions were defined using reference genome coordinates, and individual regulatory sites were assigned genomic positions and projected onto the corresponding 3′UTR space. Each site was further assigned a relative 3′UTR position (“Location at 3′UTR”), defined as the nucleotide distance from the annotated 3′UTR start site to the predicted regulatory element, enabling positional stratification along the transcript from proximal to distal regions. To evaluate regulatory architecture, genes were stratified based on total predicted regulatory site counts and classified into four categories: high hotspots (≥30 sites), medium hotspots (10–29 sites), low hotspots (3–9 sites), and basal genes (≤2 sites), reflecting increasing to minimal post-transcriptional regulatory burden, respectively. The 3′UTR hotspot region (regulatory locus) for each gene was defined as the genomic interval spanning the minimum and maximum observed regulatory site positions, representing the core clustered regulatory domain within each transcript. In addition, genes were categorized by 3′UTR structural length and regulatory architecture: very short UTRs (minimal regulatory span with ≤2 sites and highly restricted positional range), short UTRs (limited distribution of regulatory sites within compact regions), long UTRs (extended distribution of regulatory sites across broad 3′UTR intervals), and compact regulatory loci, defined as genes in which regulatory elements were tightly clustered within a narrow genomic window, versus extended regulatory loci, where sites were dispersed across multiple subregions of the 3′UTR. This integrated classification framework enabled simultaneous assessment of regulatory density and spatial organization of post-transcriptional control across complement genes, facilitating identification of regulatory hubs characterized by both high site density and extended or compact 3′UTR architectures [44,59,60,61] (Table S1).

### 4.3. miRNA Target Site Prediction Within the 3′UTRs

Predicted microRNA (miRNA) binding sites within the 3′UTRs of selected complement genes were identified using TargetScan 8.0 (https://www.targetscan.org/vert_80/; accessed on 5 April 2026) and miRBase (https://mirbase.org; accessed on 5 April 2026) [59,62]. The 3′UTR sequences of complement components, receptors, and regulatory genes were analyzed to identify potential miRNA–mRNA interactions based on canonical seed pairing and sequence conservation. Predicted binding sites were classified by seed match type: 8mer, 7mer-m8, and 7mer-A1, representing progressively weaker levels of miRNA seed complementarity. Additional parameters provided by TargetScan were used to evaluate interaction strength and regulatory relevance, including predicted dissociation constants (Kd values) and context++ scores, which integrate multiple sequence features such as site accessibility, local AU content, and distance from the stop codon to estimate the likelihood of effective miRNA-mediated repression. To prioritize biologically relevant interactions, conserved miRNA binding sites across vertebrate species were given higher priority compared with poorly conserved sites. Furthermore, cumulative weighted context++ scores were calculated to estimate the overall regulatory influence of predicted miRNAs on each complement gene. The complete set of predicted miRNA–target interactions, including seed match classification and scoring metrics, is provided in Table S2.

### 4.4. Network Construction and Cytoscape Network Analysis

For each complement gene, all predicted miRNA interactions, including conserved and poorly conserved target sites, were extracted from TargetScan output files. Individual gene-specific datasets were merged into a unified interaction matrix containing miRNA-gene regulatory pairs. Gene symbols were categorized into complement pathway components, complement receptors, regulators, and proteases.

A bipartite miRNA-gene interaction network was constructed using Cytoscape v3.10+ [63]. Genes and miRNAs served as nodes, and predicted miRNA-gene interactions were represented as edges. Node attributes included the total predicted miRNA binding burden and the number of evolutionarily conserved miRNA target sites. Edge attributes were derived from TargetScan interaction scores. The network was visualized using a force-directed layout. Node size reflected the total number of predicted miRNA binding sites, and node color corresponded to the complement pathway classification. Conserved miRNA site counts were included as an additional node attribute to identify genes subject to strong evolutionary post-transcriptional regulation (Table S3).

### 4.5. Functional Enrichment Analysis Using Cytoscape g: Profiler

Functional enrichment was performed directly in Cytoscape using the g:Profiler (g:GOSt) built-in app [64]. The curated gene list was submitted to g:Profiler to identify significantly enriched Gene Ontology (GO) Biological Processes, Molecular Functions, and Cellular Components, KEGG and Reactome pathways, and Transcription factor motifs. Significance thresholds were set at FDR < 0.05 using g: Profiler’s built-in multiple testing correction. Enriched biological processes were visualized as functional modules and integrated with the miRNA-gene regulatory network to identify biologically relevant regulatory clusters.

### 4.6. Variant Annotation, Filtering, and Disease Association Scoring

SNPs were subsequently annotated using the Ensemble Variant Effect Predictor (VEP; GRCh38). VEP provided gene mapping, variant consequence, clinical significance (ClinVar), population allele frequency, Combined Annotation Dependent Depletion (CADD) scores, and phenotype annotations. Only variants located in 3′ untranslated regions (3′UTRs) were retained, and transcript-level duplicates were collapsed to a single record per unique variant. Variant–disease relationships were derived from VEP phenotype annotations. To focus on clinically meaningful conditions, quantitative traits, laboratory measures, and nonspecific annotations were removed prior to modeling, and related phenotype terms were harmonized into broader disease categories. A composite evidence score was calculated for each variant–disease pair by summing weighted contributions from three components: (1) evidence strength based on the number and type of supporting annotation sources, (2) population rarity derived from allele frequency, and (3) predicted functional impact based on CADD PHRED score. Disease model scores (represented as total model score) were calculated using the variant counts and mean scores (Table S4).

## 5. Conclusions

### Limitations and Future Directions

In summary, this study demonstrates that miRNA regulation of the complement system is highly selective and exhibits a non-uniform distribution. Core complement components are largely devoid of conserved miRNA binding sites, whereas regulatory proteins and terminal pathway components are enriched for conserved miRNA interactions. This regulatory architecture enables rapid activation of the complement cascade while allowing fine-tuning of inflammatory responses through post-transcriptional mechanisms. These findings provide a foundation for future functional studies and suggest that miRNAs may be explored as potential targets for modulating complement-driven pathology in cardiovascular, renal, retinal and other systemic inflammatory diseases. Although computational analyses provide valuable insight into candidate regulatory variants, several important limitations of the current study must be acknowledged in interpreting these findings.

All miRNA–complement gene interactions reported here are computationally predicted; no experimental validation was performed in this study. Predicted binding sites derived from TargetScan carry inherent false-positive rates and may not fully capture in vivo targeting dynamics, which are shaped by RNA secondary structure, RNA-binding protein competition, cell-type-specific miRNA expression, and transcript isoform availability. The disease association model, while integrating allele frequency, CADD PHRED scores, and ClinVar annotations, is based entirely on existing database annotations and does not draw on independent functional or clinical datasets; variants with limited prior characterization, including many rarer rsIDs in the dataset, may therefore be systematically underscored. The analysis also does not capture the regulatory contributions of alternative polyadenylation (APA), RNA-binding proteins, or AU-rich element-mediated decay, all of which operate in the same 3′UTR space and may interact with or override miRNA-mediated control in specific inflammatory contexts. Finally, the study did not conduct formal evolutionary or population-stratified analyses, and the population-specific frequencies of the identified miR-SNPs, which may vary substantially across ancestral groups, remain uncharacterized.

These limitations define a clear agenda for future investigation. The most immediate priority is experimental validation of the predicted miRNA-complement gene interactions. Luciferase reporter assays incorporating wild-type and variant 3′UTR sequences for high-priority candidates such as CD59, CFH, CR2, and C7 would directly test whether the identified miR-SNPs alter miRNA-mediated repression. Complementary miRNA perturbation experiments, using mimics and inhibitors of recurrently targeting miRNAs such as miR-214-3p, miR-21, miR-146a, and the let-7 family, in complement-expressing cell types (hepatocytes, monocytes, endothelial cells) would establish whether these interactions are functionally operative in physiologically relevant or aberrant processes.

Importantly, integrating the candidate miR-SNPs with transcriptomic and population-level datasets will be essential for establishing biological relevance. Expression quantitative trait locus (eQTL) analyses across tissue-specific resources such as GTEx could reveal whether SNPs within predicted miRNA-binding sites are associated with changes in complement gene expression in vivo. Large-scale genomic studies examining these variants in disease cohorts, particularly for aHUS, AMD, SLE, and inflammatory cardiovascular and renal diseases, would help determine whether they contribute to disease susceptibility beyond the protein-coding variants already established in the literature.

In addition, the functional interplay between APA-driven 3′UTR remodeling and the miR-SNPs identified here warrants direct investigation. Because inflammatory signaling can reshape 3′UTR architecture by shifting polyadenylation site usage, the regulatory impact of a given miR-SNP may be present in one isoform and absent in another. Systematic APA profiling in complement-expressing cells under homeostatic versus inflammatory conditions would clarify the proportion of transcripts retaining miRNA-binding regions and the degree to which APA modulates miR-SNP accessibility.

Finally, genome-editing technologies and high-throughput functional genomics offer opportunities to investigate the regulatory consequences of noncoding complement variants in physiologically relevant models. CRISPR-mediated introduction of specific miR-SNPs into complement-expressing cell lines, combined with RNA sequencing and complement activity assays, could enable precise causal attribution of individual variants to expression-level and functional phenotypes. These approaches, taken together, would convert the candidate regulatory landscape described here into experimentally grounded insights and may ultimately contribute to the identification of novel biomarkers and miRNA-based therapeutic targets for diseases in which complement dysregulation plays a central pathogenic role.

## Data Availability

The data presented in this study utilized web-based tool for predicting the biological targets of microRNAs, and are openly available in [Targetscan 8.0] [https://www.targetscan.org/vert_80/, (accessed on 5 April 2026)].

## Supplementary Materials

The following supporting information can be downloaded at: https://www.mdpi.com/article/10.3390/ijms27135877/s1. References [3,14,17,21,22,23,24,25,26,27,28,29,30,31,32,33,34,35,36,37,38,39,40,41,42,65,66,67,68,69,70,71,72,73,74,75,76,77,78,79,80,81,82,83,84,85,86,87,88,89,90,91,92,93,94,95,96,97,98,99,100,101,102,103,104] are cited in the Supplementary Material.
