# Supplementary material for: Comprehensive Prediction Analysis of Novel Noncoding Regulatory Variants Identified in the MicroRNA Binding Regions in Complement System Genes
Source: Int J Mol Sci. 2026 Jun 30;27(13):5877. doi: 10.3390/ijms27135877 (PMC13361139; doi:10.3390/ijms27135877)
Supplement: Supplementary file 1 [file ijms-27-05877-s001.zip › Supplementary File. S1-SNPs of the coding sequences of Complement components.pdf]

Detailed Section on SNPs of the coding sequences of complement factors

Classical Pathway

The classical complement pathway is initiated by the C1 complex (C1Q–C1R–C1S) and is essential for immune complex clearance, apoptotic cell removal, and pathogen defense (1). Loss-of-function (LoF) variants in C1QA (rs121909581 (p.Gln208Ter), rs34139950 (p.Trp216Ter), C1QB (rs751172449 (p.Arg175Ter), rs1361922961), and C1QC (rs761681612 (p.Gln74ArgfsTer64), rs377549148 (p.Arg69Ter) lead to C1q deficiency, which is strongly associated with systemic lupus erythematosus (SLE) and recurrent infections due to impaired clearance of immune complexes and apoptotic debris (2, 3). Similarly, LoF variants in C1S (rs2135727106 (p.Phe378ValfsTer32), rs121909582 (p.Arg534Gly) and C2 (rs9332736, rs28934590 (p.Ser209Cys), rs151340617 (p.Gly464Arg) disrupt downstream activation of C4 and C3 convertase formation, resulting in complement deficiency syndromes characterized by increased susceptibility to infection and autoimmune manifestations (4, 5). In contrast, specific missense variants in C1R (rs1057518645 (p.Cys358Phe), rs760277934 (p.Arg301Pro), rs1057515579 (p.Leu300Pro) and C1S (rs886040975 (p.Cys294Arg), rs886040974 (p.Asp315_Val316delinsGlu) associated with periodontal Ehlers–Danlos syndrome represent gain-of-function mechanisms that promote constitutive or dysregulated protease activation within the C1 complex (6, 7). Furthermore, certain C2 variants, rs9332739 (p.Glu318=) and rs547154, are associated with reduced complement activity and confer protection against age-related macular degeneration, suggesting that partial attenuation of classical pathway activation may be protective in chronic inflammatory conditions (8-10). Known mutations in the classical complement factors are listed in Supplementary Table S5. Together, these findings demonstrate that tightly regulated classical pathway activation is critical for immune homeostasis, and both insufficient and excessive activation contribute to autoimmunity, infection susceptibility, and inflammatory disease.

Supplementary Table S5. Mutations in the coding sequences of the complement factor genes of the classical pathway.

Gene Symbol	Chrom#	Position GRCh38/hg38	REF Sequence	ALT Sequence	Variant type	Cytoband	HGVS	SNP	Phenotype
C1QA	chr1	22639291	C	T	SNV	1p36.12	C1QA(NM_015991.4):c.622C>T<br>p.(Gln208Ter)	rs12190958<br>1	C1q DEFICIENCY 1
C1QA	chr1	22639317	G	A	SNV	1p36.12	C1QA(NM_015991.4):c.648G>A<br>p.(Trp216Ter)	rs34139950	C1q DEFICIENCY 1
C1QB	chr1	22661153	C	T	SNV	1p36.12	C1QB(NM_001378156.1):c.523C>T<br>p.(Arg175Ter)	rs75117244<br>9	C1Q DEFICIENCY 2
C1QB	chr1	22659644	G	C	SNV	1p36.12	C1QB(NM_001378156.1):c.181+1G><br>C<br>p.?	rs13619229<br>61	C1Q DEFICIENCY 2
C1QB	chr1	22661354	G	A	SNV	1p36.12	C1QB(NM_001378156.1):c.724G>A<br>p.(Gly242Arg)	rs34813378	C1Q DEFICIENCY 2
C1QC	chr1	22647256	C		Deletion	1p36.12	C1QC(NM_172369.5):c.213del<br>p.(Gln74ArgfsTer64)	rs76168161<br>2	C1Q DEFICIENCY 3
C1QC	chr1	22647250	C	T	SNV	1p36.12	C1QC(NM_172369.5):c.205C>T<br>p.(Arg69Ter)	rs37754914<br>8	C1Q DEFICIENCY 3
C1QC	chr1	22644123	G	A	SNV	1p36.12	C1QC(NM_172369.5):c.100G>A<br>p.(Gly34Arg)	rs20020673<br>6	C1Q DEFICIENCY 3
C1QC	chr1	22647535	G	A	SNV	1p36.12	C1QC(NM_172369.5):c.490G>A<br>p.(Gly164Ser)	rs75259666<br>3	C1Q DEFICIENCY 3
C1R	chr12	7091533	GA	AT	Substitutio<br>n	12p13.31	C1R(NM_001733.7):c.149_150delT<br>CinsAT<br>p.(Val50Asp)	rs10575190<br>25	EHLERS-DANLOS SYNDROME, PERIODONTAL TYPE, 1
C1R	chr12	7086423	C	A	SNV	12p13.31	C1R(NM_001733.7):c.1073G>T<br>p.(Cys358Phe)	rs10575186<br>45	EHLERS-DANLOS SYNDROME, PERIODONTAL TYPE, 1
C1R	chr12	7088721	G	A	SNV	12p13.31	C1R(NM_001733.7):c.927C>T<br>p.(Cys309=)	rs76970749<br>2	EHLERS-DANLOS SYNDROME, PERIODONTAL TYPE, 1
C1R	chr12	7088853	C	G	SNV	12p13.31	C1R(NM_001733.7):c.902G>C<br>p.(Arg301Pro)	rs76027793<br>4	EHLERS-DANLOS SYNDROME, PERIODONTAL TYPE, 1

C1R	chr12	7088856	A	G	SNV	12p13.31	C1R(NM_001733.7):c.899T>C p.(Leu300Pro)	rs1057515579	EHLERS-DANLOS SYNDROME, PERIODONTAL TYPE, 1
C1R	chr12	7088721	GC…GA (11bp)	TGTCC	Substitution	12p13.31	C1R(NM_001733.7):c.917_927delinsGGACA p.(Ile306_Cys309delinsArgThr)?	rs1057518646	EHLERS-DANLOS SYNDROME, PERIODONTAL TYPE, 1
C1R	chr12	7088886	T	C	SNV	12p13.31	C1R(NM_001733.7):c.869A>G p.(Asp290Gly)	rs1057518643	EHLERS-DANLOS SYNDROME, PERIODONTAL TYPE, 1
C1S	chr12	7067704	TTTG		Deletion	12p13.31	C1S(NM_001734.5):c.1132_1135del p.(Phe378ValfsTer32)	rs2135727106	C1s DEFICIENCY
C1S	chr12	7070184	C	G	SNV	12p13.31	C1S(NM_001734.5):c.1600C>G p.(Arg534Gly)	rs121909582	C1s DEFICIENCY
C1S	chr12	7070151	C	A	SNV	12p13.31	C1S(NM_001734.5):c.1567C>A p.(Arg523=)	rs781856506	C1s DEFICIENCY
C1S	chr12	7066526	T	C	SNV	12p13.31	C1S(NM_001734.5):c.880T>C p.(Cys294Arg)	rs886040975	EHLERS-DANLOS SYNDROME, PERIODONTAL TYPE, 2
C1S	chr12	7066591	TGT		Deletion	12p13.31	C1S(NM_001734.5):c.945_947del p.(Asp315_Val316delinsGlu)	rs886040974	EHLERS-DANLOS SYNDROME, PERIODONTAL TYPE, 2
C2	chr6	31934289	TG…TC (28 bp)		Deletion	6p21.33	C2(NM_000063.6):c.841_849+19del p.?	rs9332736	C2 DEFICIENCY, TYPE I
C2	chr6	31933876	C	G	SNV	6p21.33	C2(NM_000063.6):c.626C>G p.(Ser209Cys)	rs28934590	C2 DEFICIENCY, TYPE II
C2	chr6	31943254	G	A	SNV	6p21.33	C2(NM_000063.6):c.1390G>A p.(Gly464Arg)	rs151340617	C2 DEFICIENCY, TYPE II
C2	chr6	31936027	G	A	SNV	6p21.33	C2(NM_000063.6):c.954G>A p.(Glu318=)	rs9332739	MACULAR DEGENERATION, AGE-RELATED, 14, REDUCED RISK OF
C2	chr6	31943161	G	T	SNV	6p21.33	C2(NM_000063.6):c.1360+62G>T p.?	rs547154	MACULAR DEGENERATION, AGE-RELATED, 14, REDUCED RISK OF

C4A	chr6	31996845	CT	Insertion	6p21.33	C4A(NM_007293.3):c.3694_3695dup p.(Val1233GlnfsTer75)	rs760602547	C4A DEFICIENCY

The table shows the gene names, chromosome number with the rsIDs, variant amino acid, and the phenotype.

Lectin Pathway

Genetic variation within the lectin complement pathway significantly influences host defense, inflammatory regulation, and developmental phenotypes. In MBL2, structural missense variants, rs1800450 (p.Gly54Asp), rs1800451 (p.Gly57Ala), rs5030737 (p.Arg52Cys), and rs148078249 (p.Arg52His), as well as promoter polymorphisms rs11003125, rs7096206, and rs7095891, reduce serum mannose-binding lectin levels and are strongly associated with mannose-binding lectin deficiency, recurrent infections, and immunocompromised states (11, 12). Variants in MASP1, including rs387906752 (p.His497Tyr), rs387906753 (p.Cys630Arg), and truncating mutations such as rs763360042 (p.Trp290Ter), are pathogenic and linked to 3MC syndrome due to impaired lectin pathway protease activity (13, 14). Similarly, the well-characterized MASP2 variant rs72550870 (p.Asp120Gly) leads to decreased serum MASP-2 levels and functional lectin pathway deficiency, predisposing to infection and inflammatory phenotypes (15).

Polymorphisms in FCN1 (rs2989727, rs10120023, rs10117466, rs1071583 (p.Gln275=) are primarily regulatory and associated with reduced protein levels and susceptibility to rheumatoid arthritis, leprosy, and systemic inflammation (16). In FCN2, both regulatory (rs3124952, rs3124953, rs17514136) and missense variants such as rs17549193 (p.Thr236Met) and rs7851696 (p.Ala258Pro) influence serum ficolin-2 levels and are linked to infection susceptibility and inflammatory phenotypes (17, 18). Frameshift mutations in FCN3, including rs28357092 (1637delC) and rs532781899 (p.Leu117ProfsTer6), cause complete ficolin-3 deficiency and recurrent infections (16). Additionally, pathogenic variants in collectin genes COLEC10 (rs1060505022 (p.Gly77GlufsTer66), rs773764995 (p.Cys176Ter)) and COLEC11 (rs387907075 (p.Ser169Pro), rs387907076 (p.Gly204Ser), rs1572389284 (p.Phe16SerfsTer85), rs2147963510 (p.Gly104ValfsTer29)) are associated with 3MC syndrome, highlighting the importance of lectin pathway pattern-recognition molecules in embryonic development and immune surveillance (13, 19). Genetic variants in collectin and lectin pathway genes may also influence cardiac disease, as a cohort study of 251 Brazilian patients with Chagas disease showed that polymorphisms in COLEC11 (rs7567833G>A) and MASP2 (p.D371Y) interact synergistically to enhance complement activation in response to *Trypanosoma cruzi*, thereby contributing to the development of dilated cardiomyopathy (20). These data (Supplementary Table S6) demonstrate that both coding and regulatory variants in lectin pathway genes modulate complement activation strength, with loss-of-function mutations predisposing to infection and developmental syndromes, while regulatory variants fine-tune inflammatory susceptibility.

Supplementary Table S6. Mutations in the coding sequences of the complement factor genes of the lectin pathway.

Gene symbol	Chrom#	PositionGRCh 38/hg38	REF Sequence	ALT Sequence	Variant type	Cytoband	HGVS	SNP	Phenotype
MBL2	chr10	52771475	C	T	SNV	10q21.1	MBL2(NM_001378373.1):c.161G>A p.(Gly54Asp)	rs1800450	MANNOSE-BINDING LECTIN DEFICIENCY
MBL2	chr10	52771466	C	G	SNV	10q21.1	MBL2(NM_001378373.1):c.170G>C p.(Gly57Ala)	rs1800451	MANNOSE-BINDING LECTIN DEFICIENCY
MBL2	chr10	52771482	G	A	SNV	10q21.1	MBL2(NM_001378373.1):c.154C>T p.(Arg52Cys)	rs5030737	MANNOSE-BINDING LECTIN DEFICIENCY
MBL2	chr10	52772254	G	C	SNV	10q21.1	MBL2(NM_001378373.1):c.-10+483C>G p.?	rs11003125	Recurrent infections,
MBL2	chr10	52771925	G	A	SNV	10q21.1	MBL2(NM_001378373.1):c.-9-281C>T p.?	rs7096206	Infection susceptibility,
MBL2	chr10	52771701	G	A	SNV	10q21.1	MBL2(NM_001378373.1):c.-9-57C>T p.?	rs7095891	Immunocompromised states
MBL2	chr10	52771481	C	T	SNV	10q21.1	MBL2(NM_001378373.1):c.155G>A p.(Arg52His)	rs148078249	Immunodeficiency
MASP1	chr3	187236382	G	A	SNV	3q27.3	MASP1(NM_139125.4):c.1489C>T p.(His497Tyr)	rs387906752	
MASP1	chr3	187235983	A	G	SNV	3q27.3	MASP1(NM_139125.4):c.1888T>C p.(Cys630Arg)	rs387906753	
MASP1	chr3	187235874	C	G	SNV	3q27.3	MASP1(NM_139125.4):c.1997G>C p.(Gly666Ala)	rs387906754	
MASP1	chr3	187235812	C	A	SNV	3q27.3	MASP1(NM_139125.4):c.2059G>T p.(Gly687Trp)	rs533236263	
MASP1	chr3	187253190	C	T	SNV	3q27.3	MASP1(NM_139125.4):c.870G>A p.(Trp290Ter)	rs763360042	

MASP1	chr3	187286054	C	T	SNV	3q27.3	MASP1(NM_001879.6):c.8G>A p.(Trp3Ter)	rs763048287	
MASP1	chr3	187286013	C	A	SNV	3q27.3	MASP1(NM_001879.6):c.49G>T p.(Ala17Ser)	rs746649361	
MASP1	chr3	187236420	C	T	SNV	3q27.3	MASP1(NM_001879.6):c.1303+5061G>A p.?	rs759368047	
MASP2	chr1	11046609	T	C	SNV	1p36.22	MASP2(NM_006610.4):c.359A>G p.(Asp120Gly)	rs72550870	
FCN1	chr9	134919852	C	T	SNV	9q34.3		rs2989727	RA, leprosy,Systematic inflammation
FCN1	chr9	134918413	C	T	SNV	9q34.3		rs10120023	RA, leprosy,Systematic inflammation
FCN1	chr9	134918015	G	C	SNV	9q34.3		rs10117466	RA, leprosy,Systematic inflammation
FCN1	chr9	134909954	T	C	SNV	9q34.3	FCN1(NM_002003.5):c.825A>G p.(Gln275=)	rs1071583	RA, leprosy,Systematic inflammation
FCN2	chr9	134879836	A	G	SNV	9q34.3		rs3124952	Infection susceptibility
FCN2	chr9	134887180	C	T	SNV	9q34.3	FCN2(NM_004108.3):c.707C>T p.(Thr236Met)	rs17549193	Infection susceptibility
FCN2	chr9	134880220	A	G	SNV	9q34.3		rs3124953	Heightened inflammation risk
FCN2	chr9	134880818	A	C	SNV	9q34.3	FCN2(NM_004108.3):c.-4A>C p.?	rs17514136	Infection susceptibility
FCN2	chr9	134887245	G	C	SNV	9q34.3	FCN2(NM_004108.3):c.772G>C p.(Ala258Pro)	rs7851696	Inflammatory phenotypes
FCN3			C		deletion		Ficolin-3 (FCN3) 1637delC	rs28357092	Recurrent infection/FICOLIN 3 DEFICIENCY
FCN3	chr1	27373180		G	Insertion	1p36.11	FCN3(NM_003665.4):c.349dup p.(Leu117ProfsTer6)	rs532781899	FICOLIN 3 DEFICIENCY
COLEC10/ CL-10/CL-L1	chr8	119067306	C	T	SNV	8q24.12		rs149010496	3MC SYNDROME 3

COLEC10/ CL-10/CL-L1	chr8	119091154	A		Deletion	8q24.12	COLEC10(NM_006438.5):c.228del p.(Gly77GlufsTer66)	rs1060505022	3MC SYNDROME 3
COLEC10/ CL-10/CL-L1	chr8	119105885	C	A	SNV	8q24.12	COLEC10(NM_006438.5):c.528C> A p.(Cys176Ter)	rs773764995	3MC SYNDROME 3
COLEC11	chr2	3643807	T	C	SNV	2p25.3	COLEC11(NM_024027.5):c.505T> C p.(Ser169Pro)	rs387907075	3MC SYNDROME 3
COLEC11	chr2	3643912	G	A	SNV	2p25.3	COLEC11(NM_024027.5):c.610G> A p.(Gly204Ser)	rs387907076	3MC SYNDROME 3
COLEC11	chr2	3604384	C		Deletion	2p25.3	COLEC11(NM_024027.5):c.45del p.(Phe16SerfsTer85)	rs1572389284	3MC SYNDROME 3
COLEC11	chr2	3640312	T		Deletion	2p25.3	COLEC11(NM_024027.5):c.309del p.(Gly104ValfsTer29)	rs2147963510	3MC SYNDROME 3

The table shows the gene names, chromosome number with the RSID, variant amino acid, and the phenotype.

Alternative Pathway

Genetic variability in alternative pathway genes shapes complement activation thresholds and contributes to a range of inflammatory and degenerative conditions. Coding variants in C3, including rs121909583 (p.Arg592Pro) and rs121909584 (p.Ala1094Val), are linked to aHUS through enhanced C3 activation (functional GoF), while the common polymorphism rs2230199 (p.Arg102Cys) is associated with AMD susceptibility. Variants affecting the C5 gene suggest how genetic changes can influence both disease susceptibility and therapeutic response (Supplementary Table S7). Truncating mutations, such as p.Gln19Ter and p.Arg1476Ter, have been reported in patients with complement component 5 deficiency, while missense variants, such as p.Arg885Leu and p.Arg885Cys, alter the binding epitope of the monoclonal antibody eculizumab, resulting in reduced response to anti-C5 therapy (21-23).

Variants in the key regulators of the alternative pathway amplification loop, such as CFH and CFHR family members, illustrate how small genetic changes can have significant phenotypic effects by altering alternative pathway dynamics (24). Genetic variation in alternative pathway complement genes demonstrates that both gain-of-function (GoF) and loss-of-function (LoF) mechanisms underlie inflammatory, renal, and retinal diseases. Regulatory control of complement amplification is critically mediated by CFH, where pathogenic missense and truncating variants, such as rs121913059 (p.Arg1210Cys), rs121913057 (p.Tyr899Ter), and rs796052137 (p.Arg28IlefsTer5) (frameshift), predispose to aHUS and complement factor H deficiency, reflecting defective complement inhibition (25, 26). In contrast, common polymorphisms rs1061170 (p.His402Tyr) and rs800292 (p.Val62Ile) alter CFH binding properties and are strongly associated with AMD susceptibility, illustrating how subtle functional variation modifies chronic inflammatory risk (27-29).

In CFB, variants such as rs117905900 (p.Phe286Leu) are associated with atypical hemolytic uremic syndrome (aHUS), whereas rs4151667 (p.Leu9His) and rs641153 (p.Arg32Gln) modulate complement activation and influence age-related macular degeneration (AMD) risk; truncating variants, including rs398124644 (p.Phe632CysfsTer8), cause complement factor B deficiency, consistent with LoF phenotypes (30-32). The nonsense variant rs104894667 (p.Ser42Ter) in CFD results in factor D deficiency and impaired alternative pathway activation (33-35). Similarly, mutations in CFP (e.g., rs132630258 (p.Arg161Ter), rs132630260 (p.Ser206Ter)) cause properdin deficiency, resulting in impaired stabilization of the C3. Finally, pathogenic regulatory and coding variants in CFI (e.g., rs199688124, rs121964913, rs141853578) reduce factor I activity and predispose to aHUS and AMD by impairing complement downregulation (10, 25, 31). Rare mutations of CFI in a patient (offspring of consanguineous parents) with leukocytoclastic vasculitis in the lower extremities who carried a homozygous missense mutation in the exon of the CFI (SCV000221312) revealed no increased vulnerability to bacterial infections or systemic vascular involvement (36). Glomerulonephritis with increased isolated C3 deposition (C3GN) was also attributed to a rare homozygous variant (frequency <0.0001%) in exon 10 of the CFI gene (c.1071 T>G), resulting in a replacement of an isoleucine (Ile) with a methionine (Met) at position 357 (p.Ile357Met) (37). The data support the concept that alternative pathway dysregulation, whether through excessive activation (C3, CFB GoF variants) or impaired inhibition (CFH, CFI LoF variants), is central to the pathogenesis of aHUS, AMD, complement deficiencies, and inflammatory renal disease.

Supplementary Table S7. Mutations in the coding sequences of the complement factor genes of the alternative pathway.

Gene symbol	Chromosome	PositionGRCh38/hg38	REF Sequence	ALT Sequence	Variant type	Cytoband	HGVS	SNP	Gene symbol	Phenotype
CFB	chr6	31946403	G	A	SNV	6p21.33	CFB(NM_001710.6):c.95G>A p.(Arg32Gln)	rs641153	CFB	FACTOR B FAST-SLOW POLYMORPHISM BF*FA/S
CFB	chr6	31946402	C	A	SNV	6p21.33	CFB(NM_001710.6):c.94C>A p.(Arg32=)	rs12614	CFB	FACTOR B FAST-SLOW POLYMORPHISM BF*FA/S
CFB	chr6	31946247	T	A	SNV	6p21.33	CFB(NM_001710.6):c.26T>A p.(Leu9His)	rs4151667	CFB	MACULAR DEGENERATION, AGE-RELATED, 14, REDUCED RISK OF
CFB	chr6	31948042	C	G	SNV	6p21.33	CFB(NM_001710.6):c.858C>G p.(Phe286Leu)	rs117905900	CFB	HEMOLYTIC UREMIC SYNDROME, ATYPICAL, SUSCEPTIBILITY TO, 4
CFB	chr6	31948443	A	G	SNV	6p21.33	CFB(NM_001710.6):c.967A>G p.(Lys323Glu)	rs121909748	CFB	HEMOLYTIC UREMIC SYNDROME, ATYPICAL, SUSCEPTIBILITY TO, 4
CFB	chr6	31947950	C	A	SNV	6p21.33	CFB(NM_001710.6):c.766C>A p.(Gln256Lys)	rs398123065	CFB	COMPLEMENT FACTOR B DEFICIENCY
CFB	chr6	31951180	TGTT		Deletion	6p21.33	CFB(NM_001710.6):c.1895_1898del p.(Phe632CysfsTer8)	rs398124644	CFB	COMPLEMENT FACTOR B DEFICIENCY
CFD	chr19	860686	C	A	SNV	19p13.3	CFD(NM_001928.4):c.125C>A p.(Ser42Ter)	rs104894667	CFD	COMPLEMENT FACTOR D DEFICIENCY
CFH	chr1	196747260	C	A	SNV	1q31.3	CFH(NM_000186.4):c.3643C>A p.(Arg1215=)	rs121913051	CFH	HEMOLYTIC UREMIC SYNDROME, ATYPICAL, SUSCEPTIBILITY TO, 1
CFH	chr1	196740712	G	A	SNV	1q31.3	CFH(NM_000186.4):c.2876G>A p.(Cys959Tyr)	rs121913052	CFH	COMPLEMENT FACTOR H DEFICIENCY
CFH	chr1	196740712	G	A	SNV	1q31.3	CFH(NM_000186.4):c.2876G>A p.(Cys959Tyr)	rs121913053	CFH	COMPLEMENT FACTOR H DEFICIENCY

CFH	chr1	196747189	C	T	SNV	1q31.3	CFH(NM_000186.4):c.3572C>T p.(Ser1191Leu)	rs460897	CFH	HEMOLYTIC UREMIC SYNDROME, ATYPICAL, SUSCEPTIBILITY TO, 1
CFH	chr1	196747291	AT…GA (24bp)		Deletion	1q31.3	CFH(NM_000186.4):c.3677_*4del p.?	rs796052136	CFH	HEMOLYTIC UREMIC SYNDROME, ATYPICAL, SUSCEPTIBILITY TO, 1
CFH	chr1	196677613	G	A	SNV	1q31.3	CFH(NM_000186.4):c.565G>A p.(Glu189Lys)	rs121913054	CFH	COMPLEMENT FACTOR H DEFICIENCY
CFH	chr1	196747183	T	G	SNV	1q31.3	CFH(NM_000186.4):c.3566T>G p.(Leu1189Arg)	rs121913055	CFH	HEMOLYTIC UREMIC SYNDROME, ATYPICAL, SUSCEPTIBILITY TO, 1
CFH	chr1	196690107	C	A	SNV	1q31.3	CFH(NM_000186.4):c.1204C>A p.(His402Asn)	rs1061170	CFH	MACULAR DEGENERATION, AGE-RELATED, 4, SUSCEPTIBILITY TO BASAL LAMINAR DRUSEN, INCLUDED
CFH	chr1	196673103	G	A	SNV	1q31.3	CFH(NM_000186.4):c.184G>A p.(Val62Ile)	rs800292	CFH	MACULAR DEGENERATION, AGE-RELATED, 4, SUSCEPTIBILITY TO
CFH	chr1	196690194	T	A	SNV	1q31.3	CFH(NM_000186.4):c.1291T>A p.(Cys431Ser)	rs121913056	CFH	COMPLEMENT FACTOR H DEFICIENCY
CFH	chr1	196673000	AAGA		Deletion	1q31.3	CFH(NM_000186.4):c.83_86del p.(Arg28IlefsTer5)	rs796052137	CFH	HEMOLYTIC UREMIC SYNDROME, ATYPICAL, SUSCEPTIBILITY TO, 1
CFH	chr1	196737575	T	A	SNV	1q31.3	CFH(NM_000186.4):c.2697T>A p.(Tyr899Ter)	rs121913057	CFH	HEMOLYTIC UREMIC SYNDROME, ATYPICAL, SUSCEPTIBILITY TO, 1
CFH	chr1	196676018	G	A	SNV	1q31.3	CFH(NM_000186.4):c.380G>A p.(Arg127His)	rs121913058	CFH	COMPLEMENT FACTOR H DEFICIENCY,HUS
CFH	chr1	196679671	AGA		Deletion	1q31.3	CFH(NM_000186.4):c.671_673del p.(Lys224del)	rs796052138	CFH	COMPLEMENT FACTOR H DEFICIENCY,HUS
CFH	chr1	196713817	G	A	SNV	1q31.3	CFH(NM_000186.4):c.1419G>A p.(Ala473=)	rs2274700	CFH	MACULAR DEGENERATION, AGE-RELATED, 4, SUSCEPTIBILITY TO

CFH	chr1	196727803	G	A	SNV	1q31.3	CFH(NM_000186.4):c.2237-543G>A p.?	rs1410996	CFH	MACULAR DEGENERATION, AGE-RELATED, 4, SUSCEPTIBILITY TO
CFH	chr1	196747131	C	T	SNV	1q31.3	CFH(NM_000186.4):c.3628C>T p.(Arg1210Cys)	rs121913059	CFH	COMPLEMENT FACTOR H DEFICIENCY HEMOLYTIC UREMIC SYNDROME, ATYPICAL, SUSCEPTIBILITY TO, 1, INCLUDED MACULAR DEGENERATION, AGE-RELATED, 4, SUSCEPTIBILITY TO, INCLUDED
CFH	chr1	196747131	G	A	SNV	1q31.3	CFH(NM_000186.4):c.3514G>A p.(Glu1172Lys)	rs121913060	CFH	HEMOLYTIC UREMIC SYNDROME, ATYPICAL, SUSCEPTIBILITY TO, 1
CFH	chr1	196690125	C	T	SNV	1q31.3	CFH(NM_000186.4):c.1222C>T p.(Gln408Ter)	rs121913061	CFH	BASAL LAMINAR DRUSEN
CFH	chr1	196743552	G	T	SNV	1q31.3	CFH(NM_000186.4):c.3234G>T p.(Arg1078Ser)	rs121913062	CFH	BASAL LAMINAR DRUSEN
CFH	chr1	196673968	T	C	SNV	1q31.3	CFH(NM_000186.4):c.350+6T>C p.?	rs387906550	CFH	BASAL LAMINAR DRUSEN
CFH	chr1	196747209	G	T	SNV	1q31.3	CFH(NM_000186.4):c.3592G>T p.(Glu1198Ter)	rs121913063	CFH	HEMOLYTIC UREMIC SYNDROME, ATYPICAL, SUSCEPTIBILITY TO, 1
CFH	chr1	196713905	C	G	SNV	1q31.3	CFH(NM_000186.4):c.1507C>G p.(Pro503Ala)	rs570523689	CFH	MACULAR DEGENERATION, AGE-RELATED, 4, SUSCEPTIBILITY TO
CFP	chrX	47627564	G	A	SNV	Xp11.23	CFP(NM_001145252.3):c.481C>T p.(Arg161Ter)	rs132630258	CFP	PROPERDIN DEFICIENCY, TYPE I
CFP	chrX	47628207	G	A	SNV	Xp11.23	CFP(NM_001145252.3):c.298C>T p.(Arg100Trp)	rs132630259	CFP	PROPERDIN DEFICIENCY, TYPE II

CFP	chrX	47626820	C	A	SNV	Xp11.23	CFP(NM_001145252.3): c.893G>T p.(Gly298Val)	rs28935480	CFP	PROPERDIN DEFICIENCY, TYPE I
CFP	chrX	47627290	G	C	SNV	Xp11.23	CFP(NM_001145252.3): c.617C>G p.(Ser206Ter)	rs132630260	CFP	PROPERDIN DEFICIENCY, TYPE I
CFP	chrX	47626062	A	C	SNV	Xp11.23	CFP(NM_001145252.3): c.1240T>G p.(Tyr414Asp)	rs132630261	CFP	PROPERDIN DEFICIENCY, TYPE III
C3	chr19	6718376	G	A	SNV	19p13.3	C3(NM_000064.4):c.304C>T p.(Arg102Cys)	rs2230199	C3	MACULAR DEGENERATION, AGE-RELATED, 9, SUSCEPTIBILITY TO
C3	chr19	6713251	G	A	SNV	19p13.3	C3(NM_000064.4):c.941C>T p.(Pro314Leu)	rs1047286	C3	C3 POLYMORPHISM, HAV 4-1 PLUS/MINUS TYPE C3S/C3F POLYMORPHISM
C3	chr19	6702470	C	G	SNV	19p13.3	C3(NM_000064.4):c.2354+1G>C p.?	rs112996548	C3	C3 DEFICIENCY
C3	chr19	6709754	C	G	SNV	19p13.3	C3(NM_000064.4):c.1775G>C p.(Arg592Pro)	rs121909583	C3	HEMOLYTIC UREMIC SYNDROME, ATYPICAL, SUSCEPTIBILITY TO, 5
C3	chr19	6693033	G	A	SNV	19p13.3	C3(NM_000064.4):c.3281C>T p.(Ala1094Val)	rs121909584	C3	HEMOLYTIC UREMIC SYNDROME, ATYPICAL, SUSCEPTIBILITY TO, 5
C3	chr19	6692971	C	A	SNV	19p13.3	C3(NM_000064.4):c.3343G>T p.(Asp1115Tyr)	rs121909585	C3	HEMOLYTIC UREMIC SYNDROME, ATYPICAL, SUSCEPTIBILITY TO, 5
C3	chr19	6697673	G	A	SNV	19p13.3	C3(NM_000064.4):c.2562C>T p.(Tyr854=)	rs121909586	C3	HEMOLYTIC UREMIC SYNDROME, ATYPICAL, SUSCEPTIBILITY TO, 5
C3	chr19	6678457	T	A	SNV	19p13.3	C3(NM_000064.4):c.4631-2A>T p.?	rs111595742	C3	C3 DEFICIENCY
C3	chr19	6718135	T	G	SNV	19p13.3	C3(NM_000064.4):c.463A>C p.(Lys155Gln)	rs147859257	C3	MACULAR DEGENERATION, AGE-RELATED, 9, SUSCEPTIBILITY TO
CFI	chr4	109746398	T	A	SNV	4q25		rs121964912	CFI	COMPLEMENT FACTOR I DEFICIENCY

CFI	chr4	109760523	C	T	SNV	4q25		rs199688124	CFI	COMPLEMENT FACTOR I DEFICIENCY
CFI	chr4	109746474		AT	Insertion	4q25		rs758049059	CFI	COMPLEMENT FACTOR I DEFICIENCY
CFI	chr4	109746231	G	A	SNV	4q25		rs121964913	CFI	HEMOLYTIC UREMIC SYNDROME, ATYPICAL, SUSCEPTIBILITY TO, 3
CFI	chr4	109741074	T	A	SNV	4q25		rs121964914	CFI	HEMOLYTIC UREMIC SYNDROME, ATYPICAL, SUSCEPTIBILITY TO, 3
CFI	chr4	109741008	C	A	SNV	4q25		rs121964915	CFI	HEMOLYTIC UREMIC SYNDROME, ATYPICAL, SUSCEPTIBILITY TO, 3
CFI	chr4	109760567	C	A	SNV	4q25		rs121964916	CFI	COMPLEMENT FACTOR I DEFICIENCY
CFI	chr4	109749594	G	A	SNV	4q25		rs121964917	CFI	HEMOLYTIC UREMIC SYNDROME, ATYPICAL, SUSCEPTIBILITY TO, 3
CFI	chr4	109741090	C	T	SNV	4q25		rs121964918	CFI	HEMOLYTIC UREMIC SYNDROME, ATYPICAL, SUSCEPTIBILITY TO, 3
CFI	chr4	109764664	C	T	SNV	4q25		rs141853578	CFI	MACULAR DEGENERATION, AGE-RELATED, 13, SUSCEPTIBILITY TO HEMOLYTIC UREMIC SYNDROME, ATYPICAL, SUSCEPTIBILITY TO, 3, INCLUDED
CFI	chr4	109746417	C	G	SNV	4q25		rs371432629	CFI	MACULAR DEGENERATION, AGE-RELATED, 13, SUSCEPTIBILITY TO

The table shows the gene names, chromosome number with the RSID, variant amino acid, and the phenotype.

Terminal Pathway

Genetic variation in genes encoding the terminal complement pathway components (C5, C6, C7, C8, and C9) plays a critical role in determining susceptibility to complement deficiencies and immune dysfunction. The terminal complement proteins function cooperatively to assemble the membrane attack complex (MAC), and loss of any single component disrupts MAC formation and compromises complement-mediated cytolysis. The MAC is generated by sequential binding of C5b to C6, C7, and C8, followed by polymerization of C9, which forms a transmembrane pore that lyses pathogen membranes (38, 39).

Numerous frameshift and deletion mutations in C6, including p.Ser277GlufsTer15 and p.Gln380SerfsTer7, disrupt protein structure and prevent formation of the terminal complement complex (40, 41). Mutations in C7, such as nonsense variants p.Cys750Ter and p.Cys728Ter and frameshift mutations including p.Val714LeufsTer2, have also been shown to abolish functional C7 protein production and impair complement-mediated bacterial lysis (42, 43) (Supplementary Table S8).

Defects in C8 and C9 further illustrate the importance of the terminal complement components for immune defense (44, 45). Pathogenic nonsense variants in C8B (e.g., p.Arg376Ter and p.Arg222Ter) truncate the β-subunit of C8 and disrupt assembly of the C8αβγ complex required for MAC formation. Likewise, several C9 variants, including p.Arg116Ter, p.Ser427Ter, and p.Arg154Ter, lead to C9 deficiency and impair the polymerization of C9 molecules, which are necessary for pore formation in the target membrane. Because the terminal complement pathway is the final effector stage of complement activation, deficiencies in these components significantly reduce serum bactericidal activity. Clinically, individuals carrying loss-of-function variants in terminal complement genes have a markedly increased risk of recurrent infections caused by *Neisseria* species, particularly *Neisseria meningitidis* (46, 47). These infections occur because the MAC plays a central role in complement-mediated killing of Gram-negative encapsulated bacteria. Consequently, terminal complement deficiencies are among the strongest known genetic risk factors for invasive meningococcal disease. In addition to infectious susceptibility, recent studies suggest that rare variants in terminal complement genes may also contribute to other pathological conditions, including endothelial injury and inflammatory states (48). These findings emphasize the importance of integrating genetic, functional, and clinical data to better understand the impact of complement variation on immune homeostasis and disease susceptibility (Supplementary Table S7).

Supplementary Table S8. Mutations in the coding sequences of the complement factor genes of the terminal complement complex.

Gene Symbol	Chromosome	PositionGRCh38/hg38	REF Sequence	ALT Sequence	Variant type	Cytoband	HGVS	SNP	Phenotype
C5	chr9	121050192	G	A	SNV	9q33.2	C5(NM_001735.3):c.55C>T p.(Gln19Ter)	rs121909587	COMPLEMENT COMPONENT 5 DEFICIENCY
C5	chr9	120962749	G	A	SNV	9q33.2	C5(NM_001735.3):c.4426C>T p.(Arg1476Ter)	rs121909588	COMPLEMENT COMPONENT 5 DEFICIENCY
C5	chr9	120953758	GG	C	Substitution	9q33.2	C5(NM_001735.3):c.4872_4873delCCinsG p.(Leu1625SerfsTer3)	rs387906554	COMPLEMENT COMPONENT 5 DEFICIENCY
C5	chr9	121023405	T	C	SNV	9q33.2	C5(NM_001735.3):c.1115A>G p.(Lys372Arg)	rs587776846	COMPLEMENT COMPONENT 5 DEFICIENCY
C5	chr9	121006922	C	A	SNV	9q33.2	C5(NM_001735.3):c.2404G>T p.(Val802Phe)	rs17611	
C5	chr9	120954562	T	C	SNV	9q33.2	C5(NM_001735.3):c.4763-694A>G p.?	rs2300929	
C5	chr9	120997683	C	A	SNV	9q33.2	C5(NM_001735.3):c.2654G>T p.(Arg885Leu)	rs56040400	ECULIZUMAB, POOR RESPONSE TO
C5	chr9	120997684	G	A	SNV	9q33.2	C5(NM_001735.3):c.2653C>T p.(Arg885Cys)	rs373359894	ECULIZUMAB, POOR RESPONSE TO
C6	chr5	41199857	G	A	SNV	5p13.1	C6(NM_000065.5):c.356C>T p.(Ala119Val)	rs1801033	C6 A/B POLYMORPHISM
C6	chr5	41149933	A	G	SNV	5p13.1	C6(NM_000065.5):c.2381+2T>C p.?	rs76202909	C6 DEFICIENCY, SUBTOTAL
C6	chr5	41181458		C	Insertion (homopolymer)	5p13.1	C6(NM_000065.5):c.828dup p.(Ser277GlufsTer15)	rs372345940	C6 DEFICIENCY

C6	chr5	41176505	G		Deletion	5p13.1	C6(NM_000065.5):c.1138del p.(Gln380SerfsTer7)	rs375762365	C6 DEFICIENCY
C6	chr5	41158763	C		Deletion	5p13.1	C6(NM_000065.5):c.1879del p.(Asp627ThrfsTer4)	rs61469168	C6 DEFICIENCY
C6	chr5	41201621	G		Deletion	5p13.1	C6(NM_000065.5):c.237del p.(Ile80SerfsTer43)	rs398122811	C6 DEFICIENCY
C6	chr5	41181465	T		Deletion	5p13.1	C6(NM_000065.5):c.821del p.(Gln274ArgfsTer46)	rs557023458	C6 DEFICIENCY
C7	chr5	40979809	T	A	SNV	5p13.1	C7(NM_000587.4):c.2250T>A p.(Cys750Ter)	rs121964919	C7 DEFICIENCY
C7	chr5	40979743	T	A	SNV	5p13.1	C7(NM_000587.4):c.2184T>A p.(Cys728Ter)	rs387906509	C7 DEFICIENCY
C7	chr5	40976812	TG		Deletion	5p13.1	C7(NM_000587.4):c.2140_2141del p.(Val714LeufsTer2)	rs1467298230	C7 DEFICIENCY
C7	chr5	40959520	C	A	SNV	5p13.1	C7(NM_000587.4):c.1561C>A p.(Arg521Ser)	rs121964920	C7 DEFICIENCY
C7	chr5	40931063	G	A	SNV	5p13.1	C7(NM_000587.4):c.63-1G>A p.?	rs1022194067	C7 DEFICIENCY
C7	chr5	40955428	G	A	SNV	5p13.1	C7(NM_000587.4):c.1135G>A p.(Gly379Arg)	rs121964921	C7 DEFICIENCY
C7	chr5	40958081		A	Insertion (homopolymer)	5p13.1	C7(NM_000587.4):c.1314dup p.(Leu439ThrfsTer8)	rs774370086	C7 DEFICIENCY
C7	chr5	40972442	AG		Deletion	5p13.1	C7(NM_000587.4):c.1924_1925del p.(His643ProfsTer10)	rs764871530	C7 DEFICIENCY

C7	chr5	40958230	T	A	SNV	5p13.1	C7(NM_000587.4):c.1458T>A p.(Cys486Ter)	rs121964922	C7 DEFICIENCY
C8A	chr1	56875054	C	A	SNV	1p32.2	C8A(NM_000562.3):c.277C>A p.(Gln93Lys)	rs652785	COMPLEMENT COMPONENT 8, ALPHA SUBUNIT, A/B POLYMORPHISM
C8A	chr1	56867703	G	A	SNV	1p32.2	C8A(NM_000562.3):c.171+1G>A p.?	rs775967055	COMPLEMENT C8 DEFICIENCY, TYPE I
C8A	chr1	56908003	C	A	SNV	1p32.2	C8A(NM_000562.3):c.1270C>A p.(Arg424=)	rs140856114	COMPLEMENT C8 DEFICIENCY, TYPE I
C8B	chr1	56940965	G	A	SNV	1p32.2	C8B(NM_001278543.2): c.1126C>T p.(Arg376Ter)	rs41286844	COMPLEMENT C8 DEFICIENCY, TYPE II
C8B	chr1	56949599	G	A	SNV	1p32.2	C8B(NM_001278543.2): c.664C>T p.(Arg222Ter)	rs140813121	COMPLEMENT C8 DEFICIENCY, TYPE II
C8B	chr1	56956799	G	A	SNV	1p32.2	C8B(NM_001278543.2): c.205C>T p.(Arg69Ter)	rs150022116	COMPLEMENT C8 DEFICIENCY, TYPE II
C8B	chr1	56956889	G	A	SNV	1p32.2	C8B(NM_001278543.2): c.115C>T p.(Gln39Ter)	rs146187042	COMPLEMENT C8 DEFICIENCY, TYPE II
C8B	chr1	56956824	G		Deletion	1p32.2	C8B(NM_001278543.2): c.180del p.(Asn61ThrfsTer22)	rs372968576	COMPLEMENT C8 DEFICIENCY, TYPE II
C8B	chr1	56952109	G		Deletion	1p32.2	C8B(NM_001278543.2): c.449del p.(Pro150ArgfsTer5)	rs398122867	COMPLEMENT C8 DEFICIENCY, TYPE II
C8B	chr1	56945879		CACAGCC (7bp)	Insertion	1p32.2	C8B(NM_001278543.2): c.885_891dup p.(Leu298GlyfsTer8)	rs398122868	COMPLEMENT C8 DEFICIENCY, TYPE II
C9	chr5	39341276	G	A	SNV	5p13.1	C9(NM_001737.5):c.346C>T p.(Arg116Ter)	rs121909592	C9 DEFICIENCY

C9	chr5	39342112	G	A	SNV	5p13.1	C9(NM_001737.5):c.162C>T p.(Cys54=)	rs34000044	C9 DEFICIENCY
C9	chr5	39341267	A	C	SNV	5p13.1	C9(NM_001737.5):c.355T>G p.(Cys119Gly)	rs121909593	C9 DEFICIENCY
C9	chr5	39306753	G	C	SNV	5p13.1	C9(NM_001737.5):c.1280C>G p.(Ser427Ter)	rs121909594	C9 DEFICIENCY
C9	chr5	39288785	C	A	SNV	5p13.1	C9(NM_001737.5):c.1583G>T p.(Cys528Phe)	rs2111835976	C9 DEFICIENCY
C9	chr5	39331792	G	A	SNV	5p13.1	C9(NM_001737.5):c.499C>T p.(Pro167Ser)	rs34882957	MACULAR DEGENERATION, AGE-RELATED, 15, SUSCEPTIBILITY TO
C9	chr5	39341162	G	A	SNV	5p13.1	C9(NM_001737.5):c.460C>T p.(Arg154Ter)	rs144138616	C9 DEFICIENCY

The table shows the gene names, chromosome number with the RSID, variant amino acid, and the phenotype.

Regulatory Genes and Receptors

Genetic variation in complement regulatory genes such as CD46, CD55, and CD59 plays a crucial role in maintaining immune homeostasis by preventing excessive complement activation on host cells. These genes encode membrane-bound complement regulatory proteins that protect host tissues from complement-mediated damage. Variants affecting these genes can disrupt complement regulation and contribute to a variety of immune-mediated and hematologic disorders. In the dataset analyzed, multiple variants were identified across these loci, including missense, nonsense, frameshift, in-frame deletion, and noncoding mutations associated with complement-related diseases (Supplementary Table S9).

Several pathogenic variants were observed in the CD46 gene, which encodes membrane cofactor protein (MCP), an important regulator that acts as a cofactor for factor I-mediated cleavage of C3b and C4b. Mutations in CD46 are a well-established genetic cause of atypical hemolytic uremic syndrome (aHUS), a thrombotic microangiopathy characterized by complement-mediated endothelial injury (49). For example, the missense variant p.Cys35Tyr (rs121909591) and the nonsense mutation p.Arg59Ter (rs121909590) have been reported in individuals with increased aHUS susceptibility. Additional variants identified include p.Ser240Pro (rs121909589) and splice-region mutations such as c.98-1G>C (rs1441937053), which are predicted to impair protein function or expression. Structural alterations, including the in-frame deletion p.Asp271_Ser272del (rs1255421232) and frameshift mutation p.Thr267AsnfsTer4 (rs1558056827), may further compromise MCP activity and reduce the ability of host cells to regulate complement activation (25, 50) (Supplementary Table S9).

Variants affecting CD55, which encodes decay-accelerating factor (DAF), were also identified in the dataset. CD55 inhibits complement activation by accelerating the decay of C3 and C5 convertases, thereby preventing excessive complement amplification on host cell surfaces. Several noncoding variants were associated with phenotypes involving complement dysregulation, including the Cromer blood group system Inab phenotype (rs121909603), and Dr(a-) phenotype (rs1135402914), both linked to alterations in CD55 expression. Other variants were associated with complement hyperactivation, angiopathic thrombosis, and protein-losing enteropathy (rs1135402915-rs1114167430), a condition known as CHAPLE disease (CD55 deficiency with hyperactivation of the complement system) (51, 52). Loss of CD55 expression leads to uncontrolled complement activation on endothelial and intestinal epithelial surfaces, resulting in vascular injury, thrombosis, and severe gastrointestinal manifestations (53, 54). Recent genetic and clinical studies have demonstrated that patients with CD55 deficiency may benefit from complement-targeted therapies such as eculizumab, highlighting the clinical significance of identifying pathogenic CD55 variants (51, 54, 55)

Supplementary Table S9 also includes variants in CD59, which encodes protectin (CD59), a glycosylphosphatidylinositol-anchored protein that prevents formation of the membrane attack complex by inhibiting C9 polymerization. Mutations in CD59 can lead to uncontrolled MAC formation on host cells and are associated with CD59-mediated hemolytic anemia, often accompanied by immune-mediated polyneuropathy (56-58). The frameshift mutations p.Val42SerfsTer38 (rs2133545024) and p.Asp49ValfsTer31 (rs587777149) introduce premature termination codons that likely produce truncated proteins incapable of inhibiting MAC formation. In addition, the missense variant p.Cys89Tyr (rs397514767) may disrupt disulfide bond formation, which is required for proper CD59 structure and function. Patients with inherited CD59 deficiency frequently present with chronic hemolysis, neurological symptoms, and complement-mediated tissue injury, emphasizing the essential role of CD59 in protecting host cells from complement-mediated damage (58-60).

The CR1 variant (rs2274567) represents a missense substitution (c.4973A>G; p.His1658Arg) and has been associated with resistance to severe Malaria (61). Despite altering the amino acid sequence, this variant is currently classified as benign, suggesting limited impact on protein function or disease pathogenicity. Within CR2, multiple variant types were detected, including synonymous, missense, non-coding, and nonsense variants. Two coding variants, rs1048971 (synonymous; p.Leu592=) and rs17615 (missense; p.Ser639Asn), are associated with susceptibility to Systemic Lupus Erythematosus, although both are classified as benign based on current clinical interpretation (62). In addition, a non-coding variant located in the 5′-regulatory region (rs3813946; c.-71T>A) was identified, which may influence transcriptional regulation of CR2 (63). Another non-coding splice-site variant (rs398122864; c.1225+1G>A) is classified as pathogenic and has been reported in individuals with Common Variable Immunodeficiency (CVID) (64). Furthermore, a nonsense variant (rs398122863; p.Trp766Ter) introduces a premature stop codon that could potentially truncate the CR2 protein and disrupt its normal immune function (65).

Collectively, these variants highlight the importance of membrane-bound complement regulators in maintaining immune balance. Disruption of their function can result in uncontrolled complement activation, endothelial damage, and systemic inflammatory disease. Advances in genomic sequencing and functional studies continue to expand our understanding of complement gene variation and its clinical consequences, while also supporting the development of targeted complement inhibitors as therapeutic strategies for complement-mediated disorders.

Supplementary Table S9. Mutations in the coding sequences of the genes of the regulators of complement activation.

Gene Symbol	Chrom#	Position GRCh38/hg38	REF S Sequence	ALT Sequence	Variant type	Cytoband	HGVS	SNP	Phenotype
CD46	chr1	207757020	G	A	SNV	1q32.2	CD46(NM_172351.3): c.104G>A p.(Cys35Tyr)	rs121909591	HEMOLYTIC UREMIC SYNDROME, ATYPICAL, SUSCEPTIBILITY TO, 2
CD46	chr1	207757091	C	T	SNV	1q32.2	CD46(NM_172351.3): c.175C>T p.(Arg59Ter)	rs121909590	HEMOLYTIC UREMIC SYNDROME, ATYPICAL, SUSCEPTIBILITY TO, 2
CD46	chr1	207757013	G	C	SNV	1q32.2	CD46(NM_172351.3): c.98-1G>C p.?	rs1441937053	HEMOLYTIC UREMIC SYNDROME, ATYPICAL, SUSCEPTIBILITY TO, 2
CD46	chr1	207767057	T	C	SNV	1q32.2	CD46(NM_172351.3): c.718T>C p.(Ser240Pro)	rs121909589	HEMOLYTIC UREMIC SYNDROME, ATYPICAL, SUSCEPTIBILITY TO, 2
CD46	chr1	207767148	GTGACA (6bp)		Deletion	1q32.2	CD46(NM_172351.3): c.811_816del p.(Asp271_Ser272del)	rs1255421232	HEMOLYTIC UREMIC SYNDROME, ATYPICAL, SUSCEPTIBILITY TO, 2
CD46	chr1	207767136	AC		Deletion	1q32.2	CD46(NM_172351.3): c.800_801del p.(Thr267AsnfsTer4)	rs1558056827	HEMOLYTIC UREMIC SYNDROME, ATYPICAL, SUSCEPTIBILITY TO, 2
CD55	chr1	207322542	G	A	SNV	1q32.2		rs121909603	CROMER BLOOD GROUP SYSTEM, Inab PHENOTYPE
CD55	chr1	207322544	C	A	SNV	1q32.2		rs1131690771	CROMER BLOOD GROUP SYSTEM, Inab PHENOTYPE
CD55	chr1	207326769	C	T	SNV	1q32.2		rs1135402914	CROMER BLOOD GROUP SYSTEM, Dr(a-) PHENOTYPE
CD55	chr1	207322391	G		Deletion	1q32.2		rs1135402915	COMPLEMENT HYPERACTIVATION, ANGIOPATHIC THROMBOSIS, AND PROTEIN-LOSING ENTEROPATHY
CD55	chr1	207322430	AA	CCTT	Substitution	1q32.2		rs1135402916	COMPLEMENT HYPERACTIVATION, ANGIOPATHIC THROMBOSIS, AND PROTEIN-LOSING ENTEROPATHY

CD55	chr1	207331243	G	C	SNV	1q32.2		rs1135402917	COMPLEMENT HYPERACTIVATION, ANGIOPATHIC THROMBOSIS, AND PROTEIN-LOSING ENTEROPATHY
CD55	chr1	207324558	G	A	SNV	1q32.2		rs1135402918	COMPLEMENT HYPERACTIVATION, ANGIOPATHIC THROMBOSIS, AND PROTEIN-LOSING ENTEROPATHY
CD55	chr1	207321808	C		Deletion	1q32.2		rs1114167430	COMPLEMENT HYPERACTIVATION, ANGIOPATHIC THROMBOSIS, AND PROTEIN-LOSING ENTEROPATHY
CD59	chr11	33717416	G		Deletion	11p13	CD59(NM_000611.6): c.123del p.(Val42SerfsTer38)	rs2133545024	HEMOLYTIC ANEMIA, CD59-MEDIATED
CD59	chr11	33710247	C	T	SNV	11p13	CD59(NM_000611.6): c.266G>A p.(Cys89Tyr)	rs397514767	HEMOLYTIC ANEMIA, CD59-MEDIATED, WITH IMMUNE-MEDIATED POLYNEUROPATHY
CD59	chr11	33717393	T		Deletion	11p13	CD59(NM_000611.6): c.146del p.(Asp49ValfsTer31)	rs587777149	HEMOLYTIC ANEMIA, CD59-MEDIATED, WITH IMMUNE-MEDIATED POLYNEUROPATHY
CR1	chr1	207580276	A	G	SNV	1q32.2	CR1(NM_000651.6):c.4973A>Gp.(His1658Arg)	rs2274567	MALARIA, SEVERE, RESISTANCE TO
CR2	chr1	207472977	G	A	SNV	1q32.2	CR2(NM_001006658.3):c.1776G>Ap.(Leu592=)	rs1048971	SYSTEMIC LUPUS ERYTHEMATOSUS, SUSCEPTIBILITY TO, 9
CR2	chr1	207473117	G	A	SNV	1q32.2	CR2(NM_001006658.3):c.1916G>Ap.(Ser639Asn)	rs17615	SYSTEMIC LUPUS ERYTHEMATOSUS, SUSCEPTIBILITY TO, 9
CR2	chr1	207454348	T	A	SNV	1q32.2	CR2(NM_001006658.3):c.-71T>Ap.?	rs3813946	SYSTEMIC LUPUS ERYTHEMATOSUS, SUSCEPTIBILITY TO, 9

CR2	chr1	207470103	G	A	SNV	1q32.2	CR2(NM_001006658.3):c.1225+1G>Ap.?	rs398122864	IMMUNODEFICIENCY, COMMON VARIABLE, 7 (1 patient)
CR2	chr1	207474297	G	A	SNV	1q32.2	CR2(NM_001006658.3):c.2297G>Ap.(Trp766Ter)	rs398122863	IMMUNODEFICIENCY, COMMON VARIABLE, 7 (1 patient)

The table shows the gene names, chromosome number with the RSID, variant amino acid, and the phenotype.

REFERENCES FOR THE SUPPLEMENTARY INFORMATION

1. Dunkelberger, J. R., and Song, W. C. (2010) Complement and its role in innate and adaptive immune responses. *Cell Res* **20**, 34-50
2. Botto, M., and Walport, M. J. (2002) C1q, autoimmunity and apoptosis. *Immunobiology* **205**, 395-406
3. Pickering, M. C., Fischer, S., Lewis, M. R., Walport, M. J., Botto, M., and Cook, H. T. (2001) Ultraviolet-radiation-induced keratinocyte apoptosis in C1q-deficient mice. *J Invest Dermatol* **117**, 52-58
4. Walport, M. J. (2001) Complement. First of two parts. *N Engl J Med* **344**, 1058-1066
5. Walport, M. J. (2001) Complement. Second of two parts. *N Engl J Med* **344**, 1140-1144
6. Grobner, R., Kapferer-Seebacher, I., Amberger, A., Redolfi, R., Dalonneau, F., Bjorck, E., Milnes, D., Bally, I., Rossi, V., Thielens, N., Stoiber, H., Gaboriaud, C., and Zschocke, J. (2019) C1R Mutations Trigger Constitutive Complement 1 Activation in Periodontal Ehlers-Danlos Syndrome. *Front Immunol* **10**, 2537
7. Kapferer-Seebacher, I., Pepin, M., Werner, R., Aitman, T. J., Nordgren, A., Stoiber, H., Thielens, N., Gaboriaud, C., Amberger, A., Schossig, A., Gruber, R., Giunta, C., Bamshad, M., Bjorck, E., Chen, C., Chitayat, D., Dorschner, M., Schmitt-Egenolf, M., Hale, C. J., Hanna, D., Hennies, H. C., Heiss-Kisielewsky, I., Lindstrand, A., Lundberg, P., Mitchell, A. L., Nickerson, D. A., Reinstein, E., Rohrbach, M., Romani, N., Schmuth, M., Silver, R., Taylan, F., Vandersteen, A., Vandrovcova, J., Weerakkody, R., Yang, M., Pope, F. M., Molecular Basis of Periodontal, E. D. S. C., Byers, P. H., and Zschocke, J. (2016) Periodontal Ehlers-Danlos Syndrome Is Caused by Mutations in C1R and C1S, which Encode Subcomponents C1r and C1s of Complement. *Am J Hum Genet* **99**, 1005-1014
8. Gold, B., Merriam, J. E., Zernant, J., Hancox, L. S., Taiber, A. J., Gehrs, K., Cramer, K., Neel, J., Bergeron, J., Barile, G. R., Smith, R. T., Group, A. M. D. G. C. S., Hageman, G. S., Dean, M., and Allikmets, R. (2006) Variation in factor B (BF) and complement component 2 (C2) genes is associated with age-related macular degeneration. *Nat Genet* **38**, 458-462
9. Spencer, K. L., Hauser, M. A., Olson, L. M., Schmidt, S., Scott, W. K., Gallins, P., Agarwal, A., Postel, E. A., Pericak-Vance, M. A., and Haines, J. L. (2007) Protective effect of complement factor B and complement component 2 variants in age-related macular degeneration. *Hum Mol Genet* **16**, 1986-1992
10. Skerka, C., Cochlovius, B., Hüllebrand, J. P., Goel, D., Sood, P., Pal, N., Chakravarti, A., Muth, D. R., Zeitz, O., and Zipfel, P. F. (2026) A new perspective on AMD pathogenesis: a sequential Factor H-centered view of complement dysregulation. *Front. Immunol.* **Volume 17 - 2026**
11. Eisen, D. P., and Minchinton, R. M. (2003) Impact of mannose-binding lectin on susceptibility to infectious diseases. *Clin Infect Dis* **37**, 1496-1505
12. Garred, P., Larsen, F., Madsen, H. O., and Koch, C. (2003) Mannose-binding lectin deficiency--revisited. *Mol Immunol* **40**, 73-84
13. Rooryck, C., Diaz-Font, A., Osborn, D. P., Chabchoub, E., Hernandez-Hernandez, V., Shamseldin, H., Kenny, J., Waters, A., Jenkins, D., Kaissi, A. A., Leal, G. F., Dallapiccola, B., Carnevale, F., Bitner-Glindzicz, M., Lees, M., Hennekam, R., Stanier, P., Burns, A. J., Peeters, H., Alkuraya, F. S., and Beales, P. L. (2011) Mutations in lectin complement pathway genes COLEC11 and MASP1 cause 3MC syndrome. *Nat Genet* **43**, 197-203
14. Sirmaci, A., Walsh, T., Akay, H., Spiliopoulos, M., Sakalar, Y. B., Hasanefendioglu-Bayrak, A., Duman, D., Farooq, A., King, M. C., and Tekin, M. (2010) MASP1 mutations in

patients with facial, umbilical, coccygeal, and auditory findings of Carnevale, Malpuech, OSA, and Michels syndromes. *Am J Hum Genet* **87**, 679-686

15. Stengaard-Pedersen, K., Thiel, S., Gadjeva, M., Moller-Kristensen, M., Sorensen, R., Jensen, L. T., Sjoholm, A. G., Fugger, L., and Jensenius, J. C. (2003) Inherited deficiency of mannan-binding lectin-associated serine protease 2. *N Engl J Med* **349**, 554-560
16. Munthe-Fog, L., Hummelshoj, T., Honore, C., Madsen, H. O., Permin, H., and Garred, P. (2009) Immunodeficiency associated with FCN3 mutation and ficolin-3 deficiency. *N Engl J Med* **360**, 2637-2644
17. Cedzynski, M., Atkinson, A. P., St Swierzko, A., MacDonald, S. L., Szala, A., Zeman, K., Buczylko, K., Bak-Romaniszyn, L., Wiszniewska, M., Matsushita, M., Szemraj, J., Banasik, M., Turner, M. L., and Kilpatrick, D. C. (2009) L-ficolin (ficolin-2) insufficiency is associated with combined allergic and infectious respiratory disease in children. *Mol Immunol* **47**, 415-419
18. Hummelshoj, T., Munthe-Fog, L., Madsen, H. O., Fujita, T., Matsushita, M., and Garred, P. (2005) Polymorphisms in the FCN2 gene determine serum variation and function of Ficolin-2. *Hum Mol Genet* **14**, 1651-1658
19. Munye, M. M., Diaz-Font, A., Ocaka, L., Henriksen, M. L., Lees, M., Brady, A., Jenkins, D., Morton, J., Hansen, S. W., Bacchelli, C., Beales, P. L., and Hernandez-Hernandez, V. (2017) COLEC10 is mutated in 3MC patients and regulates early craniofacial development. *PLoS Genet* **13**, e1006679
20. Hok, K. D., Rich, H. E., Shadid, A., Gunamalai, L., Weng-Mills, T., Thandavarayan, R. A., Banda, N. K., Doursout, M. F., Restrepo, M. I., and Shivshankar, P. (2025) Functional Roles of the Complement Immune System in Cardiac Inflammation and Hypertrophy. *Int J Mol Sci* **26**
21. Bouwman, H. B., and Guchelaar, H. J. (2024) The efficacy and safety of eculizumab in patients and the role of C5 polymorphisms. *Drug Discov Today* **29**, 104134
22. Nishimura, J., and Kanakura, Y. (2015) [The C5 gene polymorphism in patients with PNH]. *Rinsho Ketsueki* **56**, 103-110
23. Nishimura, J., Yamamoto, M., Hayashi, S., Ohyashiki, K., Ando, K., Brodsky, A. L., Noji, H., Kitamura, K., Eto, T., Takahashi, T., Masuko, M., Matsumoto, T., Wano, Y., Shichishima, T., Shibayama, H., Hase, M., Li, L., Johnson, K., Lazarowski, A., Tamburini, P., Inazawa, J., Kinoshita, T., and Kanakura, Y. (2014) Genetic variants in C5 and poor response to eculizumab. *N Engl J Med* **370**, 632-639
24. Rodriguez de Cordoba, S. (2023) Genetic variability shapes the alternative pathway complement activity and predisposition to complement-related diseases. *Immunol Rev* **313**, 71-90
25. Noris, M., and Remuzzi, G. (2009) Atypical hemolytic-uremic syndrome. *N Engl J Med* **361**, 1676-1687
26. Jozsi, M., Tortajada, A., Uzonyi, B., Goicoechea de Jorge, E., and Rodriguez de Cordoba, S. (2015) Factor H-related proteins determine complement-activating surfaces. *Trends Immunol* **36**, 374-384
27. Edwards, A. O., Ritter, R., 3rd, Abel, K. J., Manning, A., Panhuysen, C., and Farrer, L. A. (2005) Complement factor H polymorphism and age-related macular degeneration. *Science* **308**, 421-424
28. Haines, J. L., Hauser, M. A., Schmidt, S., Scott, W. K., Olson, L. M., Gallins, P., Spencer, K. L., Kwan, S. Y., Noureddine, M., Gilbert, J. R., Schnetz-Boutaud, N., Agarwal, A., Postel, E. A., and Pericak-Vance, M. A. (2005) Complement factor H variant increases the risk of age-related macular degeneration. *Science* **308**, 419-421

29. Lin, J. M., Tsai, Y. Y., Wan, L., Lin, H. J., Tsai, Y., Lee, C. C., Tsai, C. H., Tsai, F. J., and Tseng, S. H. (2008) Complement factor H variant increases the risk for early age-related macular degeneration. *Retina* **28**, 1416-1420
30. Black, J. R., and Clark, S. J. (2016) Age-related macular degeneration: genome-wide association studies to translation. *Genet Med* **18**, 283-289
31. Kavanagh, D., Richards, A., Noris, M., Hauhart, R., Liszewski, M. K., Karpman, D., Goodship, J. A., Fremeaux-Bacchi, V., Remuzzi, G., Goodship, T. H., and Atkinson, J. P. (2008) Characterization of mutations in complement factor I (CFI) associated with hemolytic uremic syndrome. *Mol Immunol* **45**, 95-105
32. Korzycka, J., Pawlowicz-Szlarska, E., Masajtis-Zagajewska, A., and Nowicki, M. (2022) Novel Complement Factor B Gene Mutation Identified in a Kidney Transplant Recipient with a Shiga Toxin-Triggered Episode of Thrombotic Microangiopathy. *Am J Case Rep* **23**, e936565
33. Biesma, D. H., Hannema, A. J., van Velzen-Blad, H., Mulder, L., van Zwieten, R., Kluijt, I., and Roos, D. (2001) A family with complement factor D deficiency. *J Clin Invest* **108**, 233-240
34. Sng, C. C. T., O'Byrne, S., Prigozhin, D. M., Bauer, M. R., Harvey, J. C., Ruhle, M., Challis, B. G., Lear, S., Roberts, L. D., Workman, S., Janowitz, T., Magiera, L., Doffinger, R., Buckland, M. S., Jodrell, D. J., Semple, R. K., Wilson, T. J., Modis, Y., and Thaventhiran, J. E. D. (2018) A type III complement factor D deficiency: Structural insights for inhibition of the alternative pathway. *J Allergy Clin Immunol* **142**, 311-314 e316
35. Sprong, T., Roos, D., Weemaes, C., Neeleman, C., Geesing, C. L., Mollnes, T. E., and van Deuren, M. (2006) Deficient alternative complement pathway activation due to factor D deficiency by 2 novel mutations in the complement factor D gene in a family with meningococcal infections. *Blood* **107**, 4865-4870
36. Bay, J. T., Katzenstein, T. L., Kofoed, K., Patel, D., Skjoedt, M. O., Garred, P., and Schejbel, L. (2015) Novel CFI mutation in a patient with leukocytoclastic vasculitis may redefine the clinical spectrum of Complement Factor I deficiency. *Clin Immunol* **160**, 315-318
37. Boudhabhay, I., Fremeaux-Bacchi, V., Roumenina, L. T., Moktefi, A., Goujon, J. M., Matignon, M., Caudwell, V., Audard, V., and El Karoui, K. (2019) Glomerulonephritis With Isolated C3 Deposits as a Manifestation of Subtotal Factor I Deficiency. *Kidney Int Rep* **4**, 1354-1358
38. Lokki, A., Triebwasser, M., Daly, E., Cohort, F., Kurki, M., Perola, M., Auro, K., Salmon, J., Java, A., Daly, M., Atkinson, J., Laivuori, H., and Meri, S. (2024) Rare variants in genes coding for components of the terminal pathway of the complement system in preeclampsia. *Res Sq*
39. Merle, N. S., Church, S. E., Fremeaux-Bacchi, V., and Roumenina, L. T. (2015) Complement System Part I - Molecular Mechanisms of Activation and Regulation. *Front Immunol* **6**, 262
40. Ferreira, C. S., Francisco Junior, R. D. S., Gerber, A. L., Guimaraes, A. P. C., de Carvalho, F. A. A., Dos Reis, B. C. S., Pinto-Mariz, F., de Souza, M. S., de Vasconcelos, Z. F. M., Goudouris, E. S., and Vasconcelos, A. T. R. (2023) Genetic screening in a Brazilian cohort with inborn errors of immunity. *BMC Genom Data* **24**, 47
41. Rizvi, S. M. S., S.; Dalrymple, A.; and Knutsen, A.P. (2023) A Novel Mutation Identified in Complement C6 Deficiency. *Journal of Clinical and Medical Images* **6**, 1-3
42. Kageyama, M., Hagiya, H., Ueda, Y., Ohtani, K., Fukumori, Y., Inoue, N., Wakamiya, N., Yoneda, N., Kimura, K., Nagasawa, M., Nakagami, F., Nishi, I., Sugimoto, K., and Rakugi, H. (2021) Disseminated gonococcal infection in a Japanese man with complement

7 deficiency with compound heterozygous variants: A case report. *Medicine (Baltimore)* **100**, e25265

43. Rameix-Welti, M. A., Regnier, C. H., Bienaime, F., Blouin, J., Schifferli, J., Fridman, W. H., Sautes-Fridman, C., and Fremeaux-Bacchi, V. (2007) Hereditary complement C7 deficiency in nine families: subtotal C7 deficiency revisited. *Eur J Immunol* **37**, 1377-1385
44. Bubeck, D., Roversi, P., Donev, R., Morgan, B. P., Llorca, O., and Lea, S. M. (2011) Structure of human complement C8, a precursor to membrane attack. *J Mol Biol* **405**, 325-330
45. Slade, D. J., Chiswell, B., and Sodetz, J. M. (2006) Functional studies of the MACPF domain of human complement protein C8alpha reveal sites for simultaneous binding of C8beta, C8gamma, and C9. *Biochemistry* **45**, 5290-5296
46. Bettoni, S., Maziarz, K., Stone, M. R. L., Blaskovich, M. A. T., Potempa, J., Bazzo, M. L., Unemo, M., Ram, S., and Blom, A. M. (2021) Serum Complement Activation by C4BP-IgM Fusion Protein Can Restore Susceptibility to Antibiotics in Neisseria gonorrhoeae. *Front Immunol* **12**, 726801
47. Lamb, E. R., and Criss, A. K. (2025) Terminal complement complexes with or without C9 potentiate antimicrobial activity against Neisseria gonorrhoeae. *mBio* **16**, e0014125
48. Rus, H., Cudrici, C., and Niculescu, F. (2005) C5b-9 complement complex in autoimmune demyelination and multiple sclerosis: dual role in neuroinflammation and neuroprotection. *Ann Med* **37**, 97-104
49. Liszewski, M. K., and Atkinson, J. P. (2015) Complement regulator CD46: genetic variants and disease associations. *Hum Genomics* **9**, 7
50. Liszewski, M. K., and Atkinson, J. P. (2021) Membrane cofactor protein (MCP; CD46): deficiency states and pathogen connections. *Curr Opin Immunol* **72**, 126-134
51. Can, S., Yorgun Altunbas, M., and Ozen, A. (2024) Pharmacotherapy for CD55 deficiency with CHAPLE disease: how close are we to a cure? *Expert Opin Pharmacother* **25**, 1421-1426
52. Fan, Y., Liao, J., Wang, Y., Wang, Z., Zheng, H., and Wang, Y. (2023) miR-132-3p regulates antibody-mediated complement-dependent cytotoxicity in colon cancer cells by directly targeting CD55. *Clin Exp Immunol* **211**, 57-67
53. Kaiafa, G., Papadopoulos, A., Ntaios, G., Saouli, Z., Savopoulos, C., Tsesmeli, N., Kontoninas, Z., Chatzinikolaou, A., Tsavdaridou, V., Klonizakis, I., and Hatzitolios, A. (2008) Detection of CD55- and CD59-deficient granulocytic populations in patients with myelodysplastic syndrome. *Ann Hematol* **87**, 257-262
54. Ozen, A., Comrie, W. A., Ardy, R. C., Dominguez Conde, C., Dalgic, B., Beser, O. F., Morawski, A. R., Karakoc-Aydiner, E., Tutar, E., Baris, S., Ozcay, F., Serwas, N. K., Zhang, Y., Matthews, H. F., Pittaluga, S., Folio, L. R., Unlusoy Aksu, A., McElwee, J. J., Krolo, A., Kiykim, A., Baris, Z., Gulsan, M., Ogulur, I., Snapper, S. B., Houwen, R. H. J., Leavis, H. L., Ertem, D., Kain, R., Sari, S., Erkan, T., Su, H. C., Boztug, K., and Lenardo, M. J. (2017) CD55 Deficiency, Early-Onset Protein-Losing Enteropathy, and Thrombosis. *N Engl J Med* **377**, 52-61
55. De Boer, E. C. W., Van Der Kruk, N., Pouw, R. B., Van Limbergen, J. E., and Kuijpers, T. W. (2025) A Pedigree With Complement Hyperactivation, Angiopathic Thrombosis, and Severe Protein-losing Enteropathy (CHAPLE) Disease: Variable Penetrance and Treatment With Pozelimab. *Clin Gastroenterol Hepatol* **23**, 1264-1267 e1263
56. Nevo, Y., Ben-Zeev, B., Tabib, A., Straussberg, R., Anikster, Y., Shorer, Z., Fattal-Valevski, A., Ta-Shma, A., Aharoni, S., Rabie, M., Zenvirt, S., Goldshmidt, H., Fellig, Y., Shaag, A., Mevorach, D., and Elpeleg, O. (2013) CD59 deficiency is associated with chronic

hemolysis and childhood relapsing immune-mediated polyneuropathy. *Blood* **121**, 129-135

57. Solmaz, I., Aytekin, E. S., Cagdas, D., Tan, C., Tezcan, I., Gocmen, R., Haliloglu, G., and Anlar, B. (2020) Recurrent Demyelinating Episodes as Sole Manifestation of Inherited CD59 Deficiency. *Neuropediatrics* **51**, 206-210
58. Ben-Zeev, B., Tabib, A., Nissenkorn, A., Garti, B. Z., Gomori, J. M., Nass, D., Goldshmidt, H., Fellig, Y., Anikster, Y., Nevo, Y., Elpeleg, O., and Mevorach, D. (2015) Devastating recurrent brain ischemic infarctions and retinal disease in pediatric patients with CD59 deficiency. *Eur J Paediatr Neurol* **19**, 688-693
59. Haliloglu, G., Maluenda, J., Sayinbatur, B., Aumont, C., Temucin, C., Tavil, B., Cetin, M., Oguz, K. K., Gut, I., Picard, V., Melki, J., and Topaloglu, H. (2015) Early-onset chronic axonal neuropathy, strokes, and hemolysis: inherited CD59 deficiency. *Neurology* **84**, 1220-1224
60. Yuksel, D., Oguz, K. K., Azapagasi, E., Kesici, S., Cavdarli, B., Konuskan, B., and Topaloglu, H. (2018) Uncontrolled inflammation of the nervous system: Inherited CD59 deficiency. *Neurol Clin Pract* **8**, e18-e20
61. Lorenzini, P. A., Gusareva, E. S., Ghosh, A. G., Ramli, N. A. B., Preiser, P. R., and Kim, H. L. (2023) Population-specific positive selection on low CR1 expression in malaria-endemic regions. *PLoS One* **18**, e0280282
62. Wu, H., Boackle, S. A., Hanvivadhanakul, P., Ulgiati, D., Grossman, J. M., Lee, Y., Shen, N., Abraham, L. J., Mercer, T. R., Park, E., Hebert, L. A., Rovin, B. H., Birmingham, D. J., Chang, D. M., Chen, C. J., McCurdy, D., Badsha, H. M., Thong, B. Y., Chng, H. H., Arnett, F. C., Wallace, D. J., Yu, C. Y., Hahn, B. H., Cantor, R. M., and Tsao, B. P. (2007) Association of a common complement receptor 2 haplotype with increased risk of systemic lupus erythematosus. *Proc Natl Acad Sci U S A* **104**, 3961-3966
63. Tang, Y., and Luo, Y. (2022) Identification of a novel mutation in complement receptor 2 in Chinese familial systemic lupus erythematosus. *Arch Rheumatol* **37**, 566-573
64. Christiansen, M., Offersen, R., Jensen, J. M. B., Petersen, M. S., Larsen, C. S., and Mogensen, T. H. (2019) Identification of Novel Genetic Variants in CVID Patients With Autoimmunity, Autoinflammation, or Malignancy. *Front Immunol* **10**, 3022
65. Jin H, K. Z., Jiang B, Tu M, Xu J, Cheng J, Liu W, Zhang Z, Li Y. (2023) Identification and Characterization of chCR2, a Protein That Binds Chicken Complement Component 3d. *J Immunol.* **210**, 1408-1418
